# Genomics and transcriptomics analysis reveals the mechanism of isobutanol tolerance of a laboratory evolved *Lactococcus lactis* strain

**DOI:** 10.1038/s41598-020-67635-w

**Published:** 2020-07-02

**Authors:** Jaya A. Gupta, Sagar Thapa, Madhulika Verma, Ritu Som, Krishna Jyoti Mukherjee

**Affiliations:** 1 Bioprocess and Biosystems Engineering Laboratory, School of Biotechnology, Jawaharlal Nehru University, New Delhi, 110067 India; 2 School of Computational and Integrative Sciences, Jawaharlal Nehru University, New Delhi, 110067 India

**Keywords:** Metabolic engineering, Applied microbiology

## Abstract

Isobutanol, in spite of its significant superiority over ethanol as a biofuel, remains commercially non-viable due to the non-availability of a suitable chassis which can handle the solvent toxicity associated with its production. To meet this challenge, we chose *Lactococcus lactis* which is known for its ability to handle environmental stress and carried out Adaptive laboratory evolution (ALE) in a continuous stirred tank reactor (CSTR) to evolve an isobutanol tolerant strain. The strain was grown for more than 60 days (> 250 generations) while gradually increasing the selection pressure, i.e. isobutanol concentration, in the feed. This led to the evolution of a strain that had an exceptionally high tolerance of up to 40 g/l of isobutanol even though a scanning electron microscope (SEM) study as well as analysis of membrane potential revealed only minor changes in cellular morphology. Whole genome sequencing which was done to confirm the strain integrity also showed comparatively few mutations in the evolved strain. However, the criticality of these mutations was reflected in major changes that occurred in the transcriptome, where gene expression levels from a wide range of categories that involved membrane transport, amino acid metabolism, sugar uptake and cell wall synthesis were significantly altered. Analysing the synergistic effect of these changes that lead to the complex phenotype of isobutanol tolerance can help in the construction of better host platforms for isobutanol production.

## Introduction

The growing demands for energy in the world economy, coupled with the opposing need to reduce our carbon footprint, has spurred research in the area of biofuels. Thus, major gains have been made in last decade, where systems biology approaches have been successfully combined with metabolic engineering and synthetic biology tools, to engineer microbes to produce advanced biofuels. However, we still have a long way to go before commercial production of these biofuels takes place. This is because of multiple bottlenecks in the process; one of the primary ones being the poor tolerance of these microbes for the product. This feedback inhibition by higher alcohols effectively ensures that the final product titers are so low as to make the production process economically unviable^1^.

Attempts to engineer solvent tolerance in a host have been mixed bag of success^2^. Each organism has its own intrinsic solvent tolerance level, which is genetically determined and environmentally influenced. Therefore, organic solvent tolerance is believed to be a strain-specific property^3,4^. The host response to solvent toxicity involves the induction of a complex stress response at the global level. Studies have shown that many strains of *Saccharomyces cerevisiae* exhibit restricted growth in 20 g/l butanol while lactic acid bacteria (LAB) including *Lactobacillus delbrueckii* and *Lactobacillus brevis* can grow in 30 g/l butanol. Strains of *Clostridia* are unable to tolerate more than 20 g/l butanol while *Escherichia coli* and *Zymomonas mobilis* strains showed no growth in 20 g/l butanol^5^. Thus we need to significantly increase the tolerance level of microorganisms for higher alcohols, if they are to be used as efficient alcohol producers^6,7^. To this end, various strategies have been proposed such as heterologous expression of efflux pumps, overexpression of heat shock proteins and membrane modifications^8^. The use of any single strategy has had limited success, thus a combination of all these strategies is probably essential for engineering a higher tolerance phenotype in these microbes.

It is important to understand how microbes are able to tolerate a range of alcohols and “omics” technologies have revealed lists of genes, proteins and intracellular compounds that contribute to this alcohol stress response. One strategy would be to grow cells in the presence and absence of stressors to investigate the role of specific genes related to one particular alcohol. This would help in elucidating the molecular mechanisms underlying alcohol stress tolerance thereby paving the way to designing alcohol-tolerant strains^9^. Transcriptomic analysis of *E. coli* during isobutanol stress^10^ has shown that genes involved in respiration, phosphate metabolism, and iron metabolism are differentially expressed. Network component and knockout analysis showed the involvement of several transcription factors in the isobutanol response network and the investigators proposed that malfunctioning of quinone was a trigger to isobutanol stress response^10^. In another work the transcriptome and proteome analysis of *E. coli* under n-butanol stress underscored the critical role of several mechanisms involved in cell envelope stress, oxidative stress, acid stress response, protein misfolding and efflux systems^11^. These key genes/factors involved in alleviating alcohol toxicity can be used as targets for designing alcohol-tolerant bacterial strains.

Metabolomic analysis of *E. coli* under ethanol, *n*-butanol and isobutanol stress identified several amino-acids such as isoleucine, valine, glycine, glutamate and trehalose as key metabolites that protect against stress^12^. Studies have shown that translation can be inhibited by alcohol-stress and this inhibition can be relieved by the addition of exogenous amino-acids or deletion of repressors^13^. Analysis of the *C. acetobutylicum* transcriptome under *n*-butanol found altered expression of genes from various categories such as glycolysis, amino acid biosynthesis and transport as well as oxidative stress response^14^ which is analogous to the *E. coli* response. This suggests that despite differences in membrane structure and metabolism, the mechanisms for combating n-butanol toxicity remains similar between these species.

In the present work, we decided to use the principle of directed evolution to increase the isobutanol tolerance of *L. lactis* NZ9000 strain. Given the diverse uses of LAB in the food, fermentation and dairy industry, as well as the increased repertoire of genetic engineering tools now available, LAB are ideal candidates for strain development^15^. Many strains of *lactobacilli* and *lactococci* are now generally recognized as safe (GRAS) and perfectly suited for the production of various metabolites using genome level engineering^16^. While *E. coli* has been a preferred system for the expression of recombinant proteins, LAB have now emerged as an alternative platform for the production of many recombinant products^17^. One problem with *E. coli* is the strong coupling of the biosynthetic and catabolic pathways which implies that once growth ceases, product formation rates also start to decline^18,19^. However, LAB have independent anabolic and catabolic pathways with little cross-regulation, a property that delinks product formation kinetics from growth. This facilitates sustained product formation under zero or low growth conditions, thereby allowing us to obtain product yields close to the theoretical maximum. There are many important attributes that make this strain an ideal candidate for tolerance engineering. *L. lactis* belongs to the family of lactic acid bacteria, which is known to have high tolerance for various stress conditions^20^. One of the examples of self-imposed stress is lactic acid stress that is triggered by the accumulation of lactic acid during sugar fermentation. LAB are relatively acid tolerant, even though the cell physiology is finally affected by this acid stress^21^. The cell envelope acts as the first line of defence against any environmental changes; modifications in the chemical composition of both the cell wall and membrane is triggered by stress, leading to increased cell survival^22,23^.

This work utilizes the principles of directed evolution, a laboratory process of creating biological entities with desired traits that has been successfully used for engineering microbes for various biotechnological purposes^24,25^. Often the term ‘directed evolution’ is used interchangeably with ALE when done under laboratory conditions^26^. Adaptive laboratory evolution utilizes the mechanisms of molecular evolution and accumulated adaptive changes to introduce desirable traits in microbial populations, using a consistent and long-term selection pressure that provides a growth advantage to cells with the desired phenotype. Thus, ALE has been successfully used to generate many complex phenotypes such as improving the stress resistance of *E. coli* (nutrient exhaustion, osmotic stress, heat shock)^27^, selection of quiescent *E. coli* cells with improved catabolic rates in stationary phase^28^, growth coupled production and secretion of lactic acid^29^ and enhancing the acetate resistance of *Acetobacter aceti*^30^. Evolutionary engineering of *Saccahromyces cerevisiae* has also been done to enhance its substrate utilization abilities across a range of substrates as well as its ability to handle various types of stresses^31^. Recently, high succinate producing *E. coli* strain were obtained by ALE experiment of the knockout mutant^32^. In our study, evolution was done in a continuous stirred tank reactor (CSTR) which is a very efficient bioreactor set-up for directed evolution, since it allows the cells to be grown for a very large number of generations while holding them at the desired specific growth rate (μ). In serial passaging methods, beneficial mutations are routinely lost due to drift, because only a small fraction of cells are transferred to the fresh medium while the remaining fraction is discarded. However in a CSTR the less fit cells are continuously removed and only the stress-adapted cells with improved growth rates and fitness are selected in the bioreactor^33^. One major disadvantage with chemostat based selection is strain instability, due to accumulation of spontaneous mutations resulting from this long-term culture^34^.

## Results

### Adaptive laboratory evolution for enhancing isobutanol tolerance

**Figure 1:**
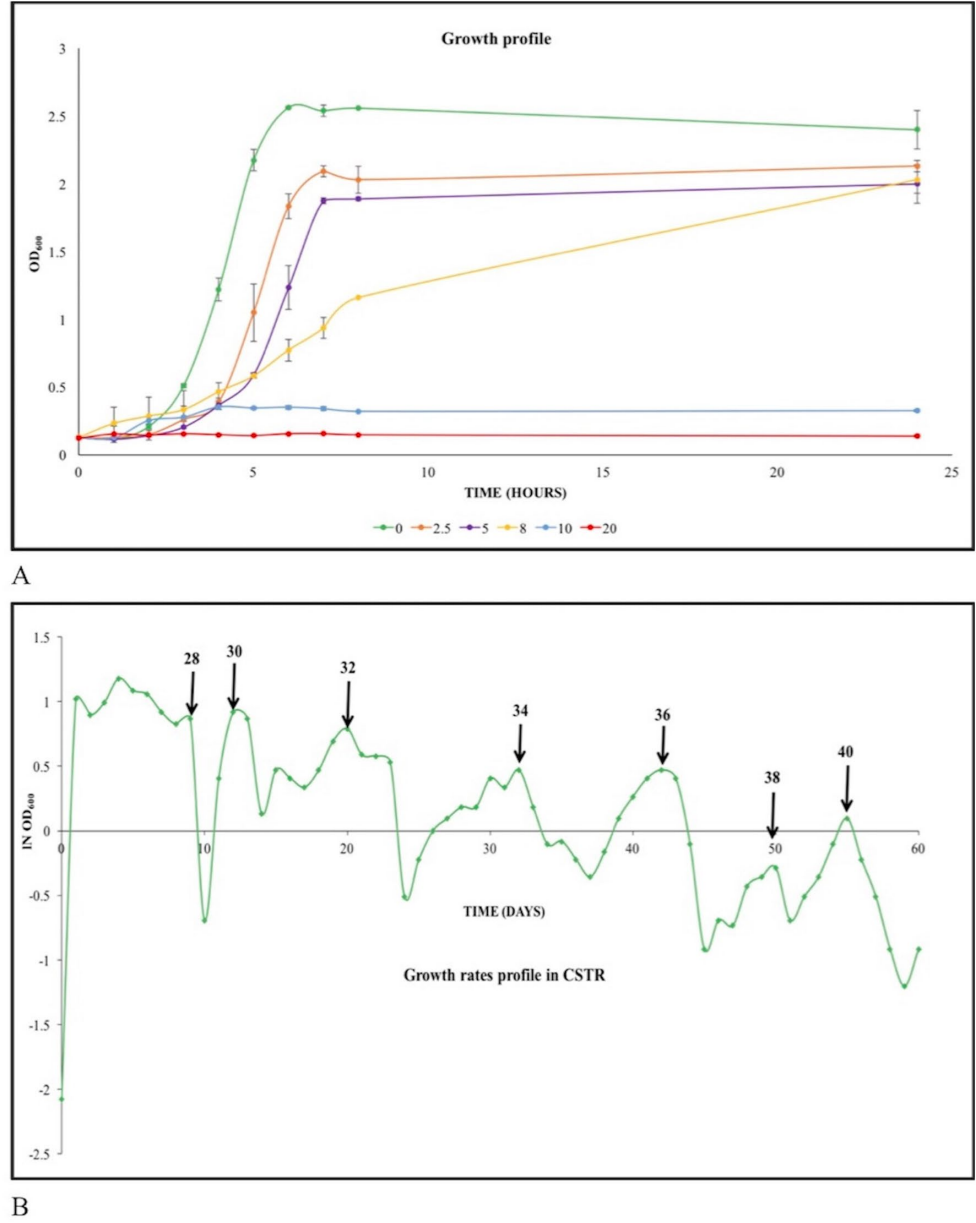
(**A**) Growth rate studies in static flasks. The native *L. lactis* NZ9000 strain was grown in GM17 media containing (0–20) g/l of isobutanol to check its level of tolerance. The graph shows a drop in both the specific growth rate as well as final OD_600_ values with increasing concentrations of isobutanol in the media. (**B**) Adaptive laboratory evolution in CSTR. The native NZ9000 strain was initially grown in GM17 broth containing 8 g/l of isobutanol in the CSTR. The arrows show the concentration of isobutanol in the feed as it was gradually increased from 28 to 40 g/l. The graph demonstrates the fast evolution of cells to reach a tolerance level of 28 g/l isobutanol followed by a slower evolution to higher concentrations where there were many instances when growth slowed down significantly as can be seen by multiple drops in the lnOD_600_ profile. At these points feed was temporarily stopped to allow biomass recovery and then restarted. Clearly each such stop-start cycle helped in the emergence of a more tolerant phenotype since no washout was subsequently observed, thus allowing us to further increase the isobutanol concentration in the feed, till the next cycle.

We first studied the growth rate of the native *L. lactis* NZ9000 strain under different concentrations (0–20 g/l) of isobutanol to determine its tolerance level. The final biomass yields as well as the specific growth rates declined with increasing isobutanol and fell drastically at concentrations greater than 8 g/l. At 20 g/l isobutanol complete cessation of growth was observed (Fig. 1A). The CSTR was therefore set up with an initial concentration of 8 g/l isobutanol in the feed. The specific growth was maintained at 0.15/ h with an initial feed rate of 115 ml/h. The incremental increase of isobutanol in the feed tank was made on the basis of changes observed in the biomass concentrations in the reactor. These changes were monitored by taking optical density (OD_600_) measurements at regular intervals. The OD profile (log scale) provides a snapshot of the multiple rounds of adaptive evolution that the cells underwent during this period (Fig. 1B). In the first round when the isobutanol concentration was increased gradually to 28 g/l within nine days, the cells adapted quickly leading to a constant biomass concentration in the bioreactor. Clearly during this period, the specific growth rate was able to match the dilution rate, in spite of the slow increase in isobutanol concentrations. However, when the concentration was increased beyond 28 g/l there was sharp decline in biomass, with the washout rate indicating an almost complete cessation of growth. Feed was temporarily stopped at this point, to allow the small sub-population of tolerant cells which may have emerged to become dominant in the culture. This point thus represents the spontaneous emergence of tolerant mutants, since adaptation is no longer sufficient to handle the high isobutanol stress. The feed rate and the increase in concentration of isobutanol were thus process variables in this experiment, which were fine tuned in order to match the rate of adaptive evolution. We thus operated the CSTR more like a turbidostat rather than a chemostat in order to prevent washout of the culture. As is clear from the biomass profile, the evolution of the final hyper-tolerant phenotype required multiple steps. Once a phenotype, tolerant to the level of isobutanol in the feed, emerged, it could grow reasonably well at that particular concentration of isobutanol. However, as the isobutanol concentration was increased further, washout was observed and feed had to be stopped to allow the emergence of an even better, more resistant phenotype. This stop-start procedure was repeated multiple times and we were successful in slowly increasing the tolerance up to 40 g/l over a period of two months. In this time, the CSTR was run continuously with brief stoppages to allow for biomass recovery.

We didn’t extend our experiment beyond 40 g/l isobutanol in the feed, as the CSTR had to be run at very low dilution rates during this period to prevent washout. We estimated that the maximum specific growth rate of the 40 g/l isobutanol evolved strain was ~ 0.05/ h, which was threefold lower than the initial dilution rates. Clearly evolution to such high tolerance levels extracts a heavy price in terms of metabolic efficiency.

### 16SrRNA polymerase chain reaction (PCR) for checking strain integrity

Since such high isobutanol tolerance has not been reported earlier, our first objective was to ensure ‘strain integrity’, so that the process of evolution of the tolerant phenotype could be validated. ‘Strain integrity’ implies that the cell population in the continuous stirred tank reactor was not contaminated or replaced by other strains. Design of species-specific primers in conjunction with universal primers allows the identification of speciation along with the presence or absence of other bacterial organisms^35^. Species-specific PCR thus remains the gold standard for confirming the genetic integrity of the strain. A polymerase chain reaction of the specific part of 16S rRNA gene was therefore done using *L. lactis* species specific forward primer and subspecies specific reverse primer for 16S rRNA^36^. The PCR products obtained from the selected (28 g/l and 40 g/l) isobutanol evolved strains along with native NZ9000 (positive control) and *E. coli* genomic DNA (negative control) as template are shown in Supplementary Fig. S1. For this PCR amplification, a proper negative control was required to show that there was no non-specific amplification and we used *E. coli* genomic DNA which was easily available in our laboratory (genomic DNA from any bacteria could have served the purpose). The absence of amplification in the negative control proved that primers were specific to *L. lactis* 16s rRNA and in the test samples we observed a 163-bp product specific to *L. lactis* subsp. *cremoris* in both the native NZ9000 as well as isobutanol evolved strains. This confirmed that the strain growing in the CSTR at higher isobutanol concentrations was indeed *L. lactis.*

### Genome sequencing of the 40 g/l isobutanol evolved strain

In order to confirm the strain genotype and identify the mutations responsible for enhanced isobutanol tolerance, the genome of the 40 g/l isobutanol evolved strain, was sequenced. Comparison with the published native NZ9000 genome sequence, allowed us to identify all the mutations that are listed in Table 1. Out of a total of 21 observed modifications, 13 of them passed QC, of which 11 were single-nucleotide polymorphisms (SNP) along with 1 single-nucleotide insertion and 1 double-nucleotide insertion. Most single-base mutations occurred in the protein coding regions, but three intergenic mutations were also observed. Among the intergenic mutations, a single-nucleotide substitution was spotted at two positions, one at Reference position: 391,489 located upstream of the LysR-family transcriptional regulator (LLNZ_02050) and another at Reference position: 2,493,226 located upstream of the ATPase, P-type (transporting), HAD superfamily, subfamily IC. The single-nucleotide insertion was observed at Reference position: 444,783 preceding the phosphoglucosamine mutase gene (LLNZ_02335). These mutant genes were upstream_gene_variants where mutations were located upstream of their CDS. One double-nucleotide insertion was detected at Reference position: 1,875,804 leading to a frameshift_variant of the phosphate transporter ATP-binding protein (LLNZ_09765). The other spotted mutations were synonymous, missense and stop_gained as we discuss in the below section. The genome sequencing also confirmed that the evolved isobutanol tolerant strain was indeed *L. lactis.* Since the 16s rRNA gene sequence showed 100% identity with the 16S rRNA of the native NZ9000 strain.

**Table 1:** Mutations identified in the 40 g/l isobutanol evolved strain (4B0 culture).

Chromosome position	Ref base	Alt base	Annotation		Gene_ID	Gene annotation
391,489	A	G	Upstream_gene_variant		LLNZ_02050	LysR family transcriptional regulator
444,783	ATTTTTTTT	ATTTTTTTTT	Upstream_gene_variant		LLNZ_02335	Phosphoglucosamine mutase
1,004,360	T	G	Synonymous_variant		LLNZ_05375	Tryptophan synthase subunit alpha
1,013,686	T	G	Synonymous_variant		LLNZ_05425	Glycine betaine-binding periplasmic protein precursor
1,191,576	C	T	Stop_gained		LLNZ_06295	d-alanine transfer protein DltD
1,238,479	C	A	Missense_variant		LLNZ_06550	Hypothetical protein
1,590,855	A	C	Synonymous_variant		LLNZ_08320	Glycosyltransferases involved in cell wall biogenesis
1,624,835	G	C	Missense_variant		LLNZ_08470	Peptidyl-prolyl cis–trans isomerase
1,875,804	CTT	CTTTT	Frameshift_variant		LLNZ_09765	Phosphate transporter ATP-binding protein
1,904,623	C	A	Missense_variant		LLNZ_09895	Exodeoxyribonuclease V alpha chain
2,058,408	A	C	Synonymous_variant		LLNZ_10680	Bifunctional N-acetylglucosamine-1-phosphate uridyltransferase/glucosamine-1-phosphate acetyltransferase
2,460,986	C	A	Missense_variant		LLNZ_12940	Predicted ATP-grasp enzyme
2,493,226	A	C	Upstream_gene_variant		LLNZ_13125	ATPase, P-type (transporting), HAD superfamily, subfamily IC

### Confirmation of the stability of evolved strains

Before proceeding further, it was important to first confirm the stability of these evolved strains. We selected three strains, tolerant to increasing levels of isobutanol (23 g/l, 30 g/l and 40 g/l) and checked their stability by initially cultivating them in the absence of isobutanol. This procedure was as follows: cells were first streaked onto agar plates in the absence of isobutanol where the emergence of an isolated colony from a single cell represents around 12–16 generations of growth. Single colonies from different plates were inoculated in culture tubes, again without isobutanol, and grown overnight in a 30 °C incubator. Secondary cultures, containing isobutanol at different concentrations, were then inoculated using this overnight primary culture, and growth rates were monitored (Supplementary Fig. S2A,D). This procedure removed selection pressure for more than 16–18 generations following which the cells were tested for their ability to grow in the presence of isobutanol. The non-evolved wild type cells served as control. We observed that the cells retained their tolerant phenotype and grew well at higher isobutanol concentrations, where as expected the most tolerant strain showed the least drop in growth at progressively higher isobutanol concentrations. The maximum specific growth rates of these different cell types, grown under increasing isobutanol concentrations is shown in Table 2. It is important to note that in spite of repeated experiments the maximum specific growth rate for the 30 g/l isobutanol evolved strain was always superior to the 40 g/l isobutanol evolved strain when they were cultured in the presence of 24 g/l isobutanol. This phenomenon of lower μ_max_ could be due to “fitness cost” imposed on the 40 g/l isobutanol evolved strain. Clearly the ability of this strain to grow in the presence of much higher isobutanol concentrations would have been achieved by a higher degree of cellular reprogramming which impacts on optimal performance and the strain would also be carrying a correspondingly larger number of mutations.

**Table 2:** Maximum specific growth rates (μ_max_) h^−1^ of native NZ9000 and isobutanol evolved strains exposed to different percentages of isobutanol in GM17 media.

Strains	GM17 media	GM17 + 8 g/l isobutanol	GM17 + 16 g/l isobutanol	GM17 + 24 g/l isobutanol	GM17 + 32 g/l isobutanol	GM17 + 40 g/l isobutanol
NZ9000	0.69	0.16	Negligible	Negligible	Negligible	Negligible
23 g/l isobutanol evolved	0.514	0.404	0.252	0.063	Negligible	Negligible
30 g/l isobutanol evolved	0.517	0.4	0.33	0.231	0.076	Negligible
40 g/l isobutanol evolved	0.585	0.484	0.359	0.19	0.108	0.03

### Cell morphology studies using SEM

The next step was to locate the reasons for improved tolerance in terms of cell morphology. Surface changes in cell wall or cell membrane structure act as the first line of defence against environmental stress^37^. While, membrane level changes has been observed in gram-negative bacteria leading to alcohol tolerance^38^, little is known about the mechanisms of organic solvent tolerance in gram-positive bacteria. Several mechanisms have been proposed^39^, among which the most common is alterations in cell morphology and filamentous growth which has also been experimentally observed in solvent tolerant gram-positive bacteria due to organic solvent stress^40^.

**Figure 2:**
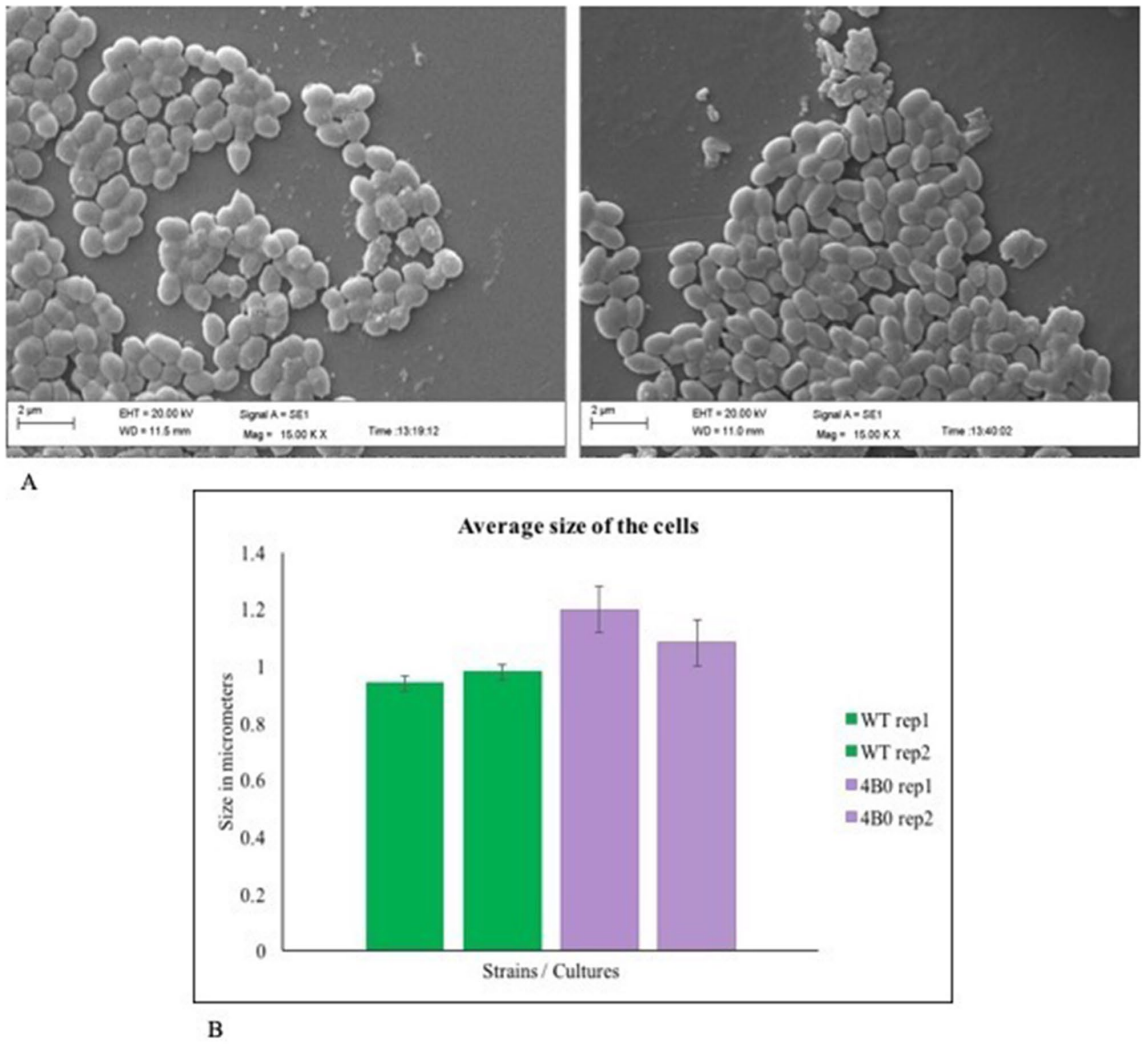
(**A**) Scanning electron micrographs. Native NZ9000 (left panel) and 4B0 culture (right panel): cells were grown in GM17 media without isobutanol to similar ODs and observed under equal magnification (15KX). (**B**) Average cell size calculated from SEM images. Graph showing a marginal increase in size of isobutanol evolved cells: average cell size (in duplicate) of native NZ9000 cells (WT rep1, WT rep2) and 40 g/l isobutanol evolved 4B0 cells (4B0 rep1, 4B0 rep2) calculated from SEM images.

We used scanning electron microscopy (SEM) to get information about the changes that may have occurred in surface morphology and size of the cells, in order to counter isobutanol toxicity. Similar studies has been done earlier where changes were observed in the cell surface structures of butanol and isobutanol tolerant strains^41^. We observed that the size of the maximal isobutanol tolerant strain (4B0 culture i.e. 40 g/l isobutanol evolved strain grown in the absence of isobutanol) had increased by around 19% (and also become more rod shaped than cocci) but not deviated significantly compared to native NZ9000 (Fig. 2A,B). A possible explanation for this increase (as we show later) is that this is an effect of isobutanol toxicity on the cell division machinery genes.

### Membrane potential analysis

An earlier study has shown that membrane potential regulates the distribution of several conserved cell division proteins such as MinD, FtsA, and the bacterial cytoskeletal protein MreB^42^. In another study conducted by Dills et al., 1980 it was found that membrane potential plays an important role in nutrient transport processes. We performed a similar study where we qualitatively compared the difference in fluorescence quenching of the membrane potential (ΔΨ) probe, 3,3′-dipropylthiadicarbocyanine iodide (DiSC3), between the native and the evolved strain. The distribution of DiSC3 over the cytoplasmic membrane and soluble phase is sensitive to changes in membrane potential, with more probe molecules tending to dissolve in the membrane with increasing (ΔΨ), leading to dye aggregation and fluorescence quenching^43,44^.

**Figure 3:**
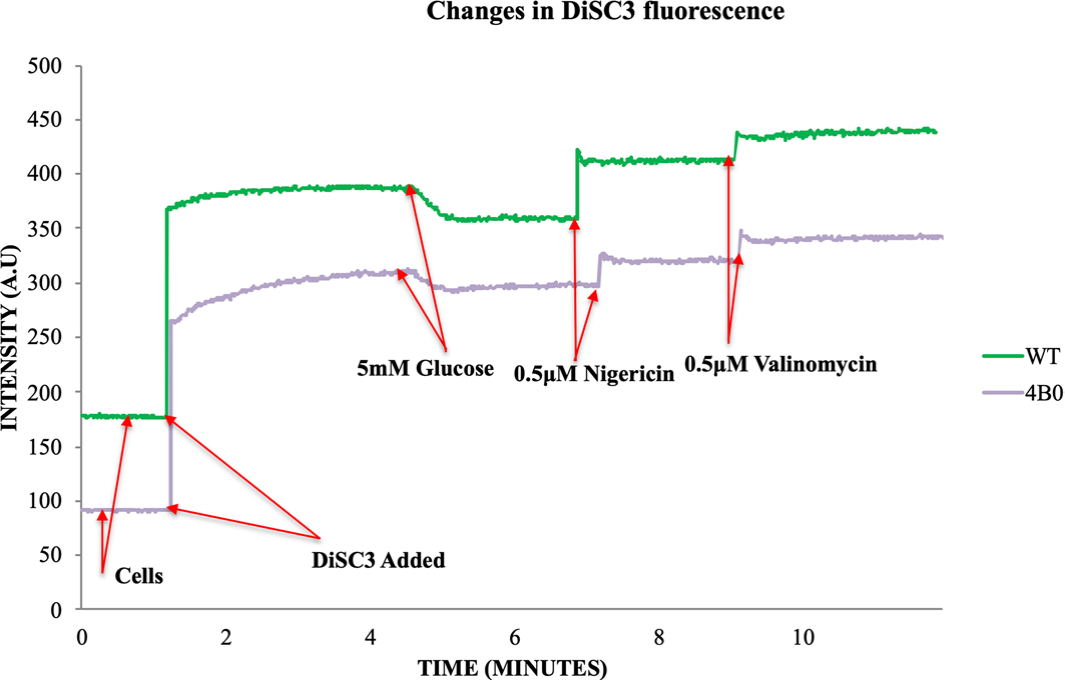
Generation of membrane potential shown by fluorescence traces of DiSC3. Changes recorded in DiSC3 fluorescence upon depolarization and hyperpolarization in whole cells. The similar change in fluorescence between native NZ9000 and 4B0 culture shows that there was no significant alteration in the membrane potential of the evolved strain.

The change in DiSC3 fluorescence caused by depolarization and hyperpolarization was recorded for both wild type and the evolved cells (Fig. 3). The proton motive force (PMF) is composed of ΔpH and ΔΨ. To estimate the contribution of the pH gradient to PMF, nigericin was added (K^+^/H^+^ exchanger) to convert the ΔpH into ΔΨ. Moreover, the addition of valinomycin (K^+^ ionophore) plus nigericin collapsed the ΔΨ entirely. The main proton pump responsible for PMF generation in *L. lactis* is the F1-Fo ATPase, which pumps protons at the expense of metabolic ATP^45^. From the observed difference in the pattern of dye fluorescence we could infer that there was only a slight difference in the magnitude of the ΔΨ change between the wild type and the isobutanol evolved cells. These experiments were repeated multiple times to confirm that the observed pattern remained the same, even though the intensity plotted on the Y-axis tended to vary. When equal amounts of nigericin was added, the evolved 4B0 culture showed a lesser response compared to the wild type cells. However, a comparison of these results with earlier reports suggests that these changes in membrane potential were too small to be a major factor in the evolution of isobutanol tolerance.

### Transcriptomic analysis of 40 g/l isobutanol evolved strain

We therefore decided to study the transcriptional changes which would have played a critical role in the development of isobutanol tolerance. This study could also be a useful guide in the design of a isobutanol tolerant phenotype through reverse engineering. We analysed the global transcriptomic profile of the isobutanol evolved strain grown both in the presence and absence of isobutanol with the native NZ9000 serving as control.

After performing the standard quality control checks of the reads using FastQC we proceeded for further analysis. The Fragments Per Kilobase Million (FPKM) values were calculated from paired-end RNA-sequencing data where two reads can correspond to a single fragment, or, if one read in the pair did not map, one read can correspond to a single fragment. FPKM thus provides the probabilistic estimates of the abundance of gene transcripts across all samples.

In order to assess the magnitude of random variation, we compared data between biological duplicates of the wild type NZ9000 strain. The correlation between abundance measurements of the gene transcripts between duplicates was computed from the log FPKM values (Supplementary Fig. S3). We observed that there was some biological variability in the data, but the correlation obtained was fairly good. Since, we were primarily interested in the genes that were maximally up and downregulated compared to control, it was important to demonstrate that these changes were statistically significant. Clearly, given the much smaller level of variation observed between the replicates, the log FC values of these maximally up and downregulated genes were significantly higher.

### Gene transcript profiles in 4B0 culture

Transcriptomic analysis showed that the 4B0 culture (40 g/l isobutanol evolved strain grown in the absence of isobutanol) had 578 differentially expressed genes compared to the native NZ9000 (WT). Genes having log fold change values of at least 2 (log_2_ fold change ≥ 2.5 or log_2_ fold change ≤  − 2.5) and false-discovery-rate (FDR) corrected P value < 0.05 were considered as differentially expressed genes. This was an unexpected and interesting result given that the number of genomic mutations were much smaller. Evidently mutations in regulatory regions must have triggered a cascade of events leading to such a drastic change in the transcriptomic landscape of the evolved strain. We therefore selected a few affected genes (from the set of differentially expressed genes) and used them to check the transcriptomic data by Reverse transcription quantitative PCR (RT-qPCR). We compared the log fold changes in gene expression obtained from RNA-seq as well as from RT-qPCR (Table 3) and observed a similar pattern, even though the actual fold change values did not match that well. The RT-qPCR experiments were done essentially to validate the RNA-seq data and confirm that the genes identified were actually up or down regulated. This was clearly established even though the actual numerical values of the fold changes were different. This is expected because of the difference in methodologies and has been reported previously as well^46^. However we do see a reasonable correlation between these two data sets (R^2^ = 0.73). It is relevant to point out that in comprehensive studies comparing these two data sets the best correlations obtained were in the range of (0.8–0.85)^46^ and these correlations can decline when smaller or specific data sets are chosen. We can safely conclude that the quantitative differences observed between these two methods of evaluating fold changes reflect on the essential limitations of the techniques and not on actual differences. More importantly taken together they confirm that we can safely use the broad categories of up or down regulated for the chosen genes. We therefore restricted our transcriptomic analysis to looking at patterns of up and downregulation rather than specific fold change values.

**Table 3:** Comparative fold changes of transcripts calculated from RNA-seq and RT-qPCR.

S. No	Locus_tag	Annotation	LogFC RNA-seq	LogFC RT-qPCR
1	LLNZ_07350	Sucrose specific PTS system IIBC component	5.48	2
2	LLNZ_02240	Carbon starvation protein A	4.3	2.45
3	LLNZ_09555	Multidrug resistance ABC transporter ATP-binding and permease protein	2.84	4.1
5	LLNZ_05785	Pyruvate kinase	− 5.5	− 1.85
6	LLNZ_11895	Non-heme iron binding ferritin	− 7.29	− 1

The results show that RT-qPCR and RNA-seq experiments identified the same genes as up or downregulated but the numerical fold change values were quite different.

A cursory look showed that these differentially expressed genes were spread all over the genome. A major concern was that a large fraction of the up and downregulated genes belonged to unknown pathways making it difficult to draw firm conclusions. However, if we focus only on the known pathways we see that a significant proportion of the top100 upregulated genes belonged to amino acid metabolism and membrane transport pathways while a large fraction of the top100 downregulated genes was part of translation and carbohydrates metabolism (Tables 4, 5). Clearly the emergence of isobutanol tolerance correlated with changes in gene expression levels from a range of pathways. In our analysis, we therefore decided to look at the transcriptional regulators (which control large sets of genes) rather than focus on any one particular pathway.

**Table 4:** Maximally upregulated genes in 4B0 culture.

S. No	Locus_tag	Annotation	Pathway
1	LLNZ_00105	PTS system, mannitol-specific IIBC component	Fructose and mannose metabolism
2	LLNZ_00455	*O*-acetylhomoserine sulfhydrylase	Cysteine and methionine metabolism
3	LLNZ_05825	Cell wall surface anchor family protein	Quorum sensing
4	LLNZ_05370	Tryptophan synthase subunit beta	Biosynthesis of amino acids
5	LLNZ_05015	Phosphoribosylformylglycinamidine synthase II	Purine metabolism
6	LLNZ_00730	Argininosuccinate synthase	Arginine biosynthesis
7	LLNZ_06600	Acetolactate synthase catalytic subunit	Valine, leucine and isoleucine biosynthesis
8	LLNZ_07350	Sucrose-specific PTS system IIBC component	Phosphotransferase system (PTS)
9	LLNZ_01385	Fructose-1,6-bisphosphatase class 3	Glycolysis/gluconeogenesis
10	LLNZ_05080	Phosphoribosylaminoimidazole synthetase	Purine metabolism
11	LLNZ_10425	ABC-type oligopeptide transport system, periplasmic component	Quorum sensing
12	LLNZ_05120	Bifunctional phosphoribosylaminoimidazolecarboxamide formyltransferaseIMP cyclohydrolase	Purine metabolism
13	LLNZ_05325	Anthranilate phosphoribosyltransferase	Phenylalanine, tyrosine and tryptophan biosynthesis
14	LLNZ_01920	Dipeptide transport ATP-binding protein dppD	ABC transporters
15	LLNZ_11520	Galactokinase	Galactose metabolism
16	LLNZ_05020	Amidophosphoribosyltransferase	Purine metabolism
17	LLNZ_01800	Putative cobalt ABC transporter ATP-binding protein	ABC transporters
18	LLNZ_03855	Neopullulanase	Starch and sucrose metabolism
19	LLNZ_09415	Menaquinone biosynthesis related protein	Ubiquinone and other terpenoid-quinone biosynthesis
20	LLNZ_02350	Beta-glucoside-specific PTS system IIABC component	Phosphotransferase system (PTS)
21	LLNZ_01670	Nisin biosynthesis two-component system, sensor histidine kinase NisK	Two-component system
22	LLNZ_06705	ATP phosphoribosyltransferase regulatory subunit	Histidine metabolism
23	LLNZ_02765	Acetylornithine deacetylase	Arginine biosynthesis

These genes are mostly involved in amino acid metabolism and membrane transport. The genes are arranged in order of their logFC values, with the top most gene having the maximum logFC value.

**Table 5:** Maximally downregulated genes in 4B0 culture.

S. No	Locus_tag	Annotation	KEGG pathway
1	LLNZ_04655	50S ribosomal protein L31 type B	Translation
2	LLNZ_13115	Glyceraldehyde 3-phosphate dehydrogenase	Glycolysis/gluconeogenesis
3	LLNZ_08605	50S ribosomal protein L19	Translation
4	LLNZ_09890	30S ribosomal protein S20	Translation
5	LLNZ_08905	30S ribosomal protein S1	Translation
6	LLNZ_03175	Phosphopyruvate hydratase	Glycolysis/gluconeogenesis
7	LLNZ_01555	30S ribosomal protein S4	Translation
8	LLNZ_10460	50S ribosomal protein L20	Translation
9	LLNZ_10690	30S ribosomal protein S15	Translation
10	LLNZ_07340	Triosephosphate isomerase	Glycolysis/gluconeogenesis
11	LLNZ_11185	Fructose-bisphosphate aldolase	Glycolysis/gluconeogenesis
12	LLNZ_12205	50S ribosomal protein L15	Translation
13	LLNZ_13150	30S ribosomal protein S9	Translation
14	LLNZ_12300	50S ribosomal protein L23	Translation
15	LLNZ_02775	Enoyl-(acyl carrier protein) reductase	Fatty acid biosynthesis
16	LLNZ_13155	50S ribosomal protein L13	Translation
17	LLNZ_12245	30S ribosomal protein S14	Translation
18	LLNZ_12790	30S ribosomal protein S6	Translation
19	LLNZ_02805	4-oxalocrotonate tautomerase	Benzoate degradation
20	LLNZ_07675	50S ribosomal protein L27	Translation
21	LLNZ_07685	50S ribosomal protein L21	Translation
22	LLNZ_01065	50S ribosomal protein L28	Translation
23	LLNZ_08170	Preprotein translocase subunit SecG	Protein export
24	LLNZ_04795	30S ribosomal protein S16	Translation
25	LLNZ_12340	preprotein translocase subunit SecE	Quorum sensing
26	LLNZ_05790	l-lactate dehydrogenase	Glycolysis/gluconeogenesis
27	LLNZ_01310	Phosphoglycerate kinase	Glycolysis/gluconeogenesis
28	LLNZ_05785	Pyruvate kinase	Glycolysis/gluconeogenesis
29	LLNZ_01860	Phosphoglyceromutase	Glycolysis/gluconeogenesis
30	LLNZ_00975	Cellobiose-specific PTS system IIC component	Phosphotransferase system (PTS)
31	LLNZ_03795	PTS system, mannose-specific IIAB components	Fructose and mannose metabolism
32	LLNZ_00645	Phosphoenolpyruvate-protein phosphotransferase	Phosphotransferase system (PTS)
33	LLNZ_05195	GMP synthase	Purine metabolism
34	LLNZ_06080	Branched-chain amino acid aminotransferase	Cysteine and methionine metabolism
35	LLNZ_03790	Mannose-specific PTS system component IIC	Fructose and mannose metabolism

These genes are mostly involved in translation and carbohydrates metabolism. The genes are arranged in order of their logFC values, with the top most gene having the minimum logFC value.

We observed that many transcriptional regulators were differentially expressed, these could be responsible for the change in expression of multiple downstream genes. For example—the CodY transcriptional repressor (LLNZ_00910) globally controls nitrogen metabolism such as proteins involved in amino acid biosynthesis and in functions related to nitrogen supply^47^. This was significantly downregulated in the 4B0 culture (logFC =  − 5.4). We correspondingly found that several genes, which are known to be affected by the downregulation of CodY, like argininosuccinate synthase, ABC-type oligopeptide transport system, dihydrodipicolinate reductase etc. were significantly upregulated (Table 6).

**Table 6:** CodY transcriptional repressor controlled gene transcripts that were upregulated in 4B0 culture due to CodY downregulation.

S. No	Locus_tag	Annotation	LogFC	KEGG pathway
1	LLNZ_00730	Argininosuccinate synthase	5.7	Arginine biosynthesis
2	LLNZ_10425	ABC-type oligopeptide transport system, periplasmic component	5.12	Peptide uptake
3	LLNZ_05325	Anthranilate phosphoribosyltransferase	4.98	Phenylalanine, tyrosine and tryptophan biosynthesis
4	LLNZ_05020	Amidophosphoribosyltransferase	4.77	Alanine, aspartate and glutamate metabolism
5	LLNZ_05715	Dihydroorotate dehydrogenase electron transfer subunit	4.29	Pyrimidine metabolism
6	LLNZ_05720	Dihydroorotate dehydrogenase 1B	4.19	Pyrimidine metabolism
7	LLNZ_09690	Aminopeptidase P	4.12	Peptide degradation
8	LLNZ_02900	Phosphoserine aminotransferase	3.35	Glycine, serine and threonine metabolism
9	LLNZ_03630	Oligopeptide transport ATP-binding protein oppF	3	Peptide uptake
10	LLNZ_04835	Dihydrodipicolinate reductase	2.1	Lysine biosynthesis

**Figure 4:**
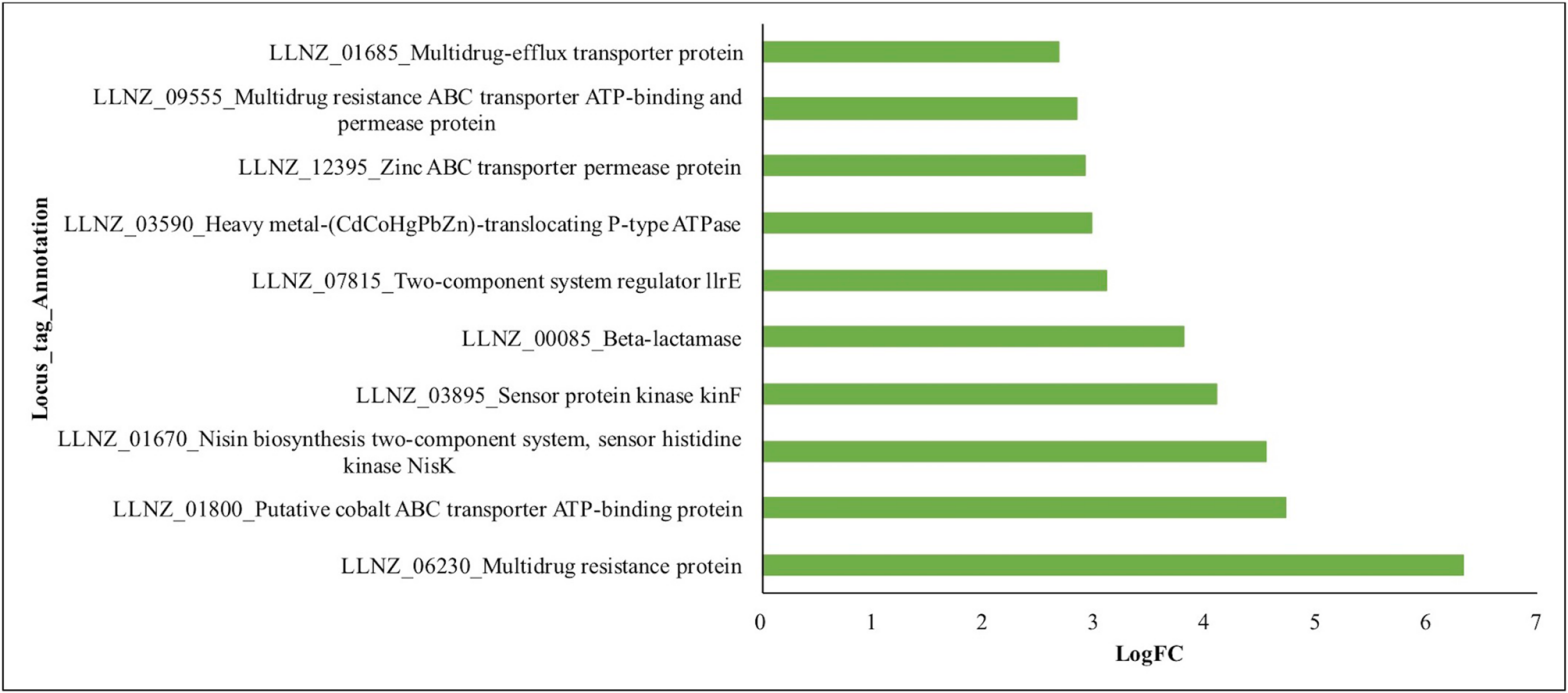
PadR, MarR and TetR family transcriptional regulator controlled genes. Gene transcripts upregulated in 4B0 culture due to upregulation of MarR and TetR that are involved in multidrug resistance, heavy metals stress and detoxification.

The PadR transcriptional regulator (LLNZ_01700) that is involved in the regulation of genes required for detoxification of phenolic acid compounds^48^ was upregulated (logFC = 1.5). Another related transcriptional regulator found upregulated was MarR (LLNZ_01690, logFC = 3.75) which is involved in resistance to multiple antibiotics. The TetR transcriptional regulator (LLNZ_01285) that controls the regulation of efflux pumps and transporters involved in antibiotic resistance and tolerance to toxic chemicals^49^ was also upregulated (logFC = 4.21). As expected, we observed that many gene transcripts that are under the control of these transcriptional regulators were also upregulated (Fig. 4). Thus, the evolution of the tolerant phenotype was the result of changes in the global regulatory machinery which then had a cascading effect on multiple downstream genes.

The expression of the cell division machinery genes was also altered (Table 7). These results correlate with the altered shape as well as increase in the size of the 4B0 cells as observed under SEM. For e.g. the increased expression (logFC = 3.4) of rod-shape determining protein MreD (LLNZ_12955) correlates with the observed change in shape of 4B0 cells. A possible advantage of the increase in size would be a lowered surface area to volume ratio which would help in countering isobutanol toxicity.

**Table 7:** Downregulated genes in 4B0 culture which are involved in cell division machinery.

S. No	Locus_tag	Annotation	LogFC
1	LLNZ_10610	Cell division protein FtsA	− 2.8
2	LLNZ_10605	Cell division protein FtsZ	− 3.7
3	LLNZ_00100	Putative cell division protein	− 2.75
4	LLNZ_04000	Putative DivIVA cell division initiation protein	− 4.6

The native sugar transport system components (cellobiose-specific phosphotransferase system (PTS) system IIC (LLNZ_02285), mannose-specific PTS system IIC (LLNZ_03790) etc.) which are usually active in normal cells were downregulated, whereas the other cellobiose-specific PTS system IIC component (LLNZ_00975) was found to be upregulated. The usually repressed sugar transport system components like the (PTS system, mannitol-specific IIBC (LLNZ_00105), glycerol uptake facilitator (LLNZ_05675), beta-glucoside-specific PTS system IIABC (LLNZ_02350), sucrose-specific PTS system IIBC (LLNZ_07350) etc.) were also found to be highly upregulated in our study. This demonstrates that the cell had reprogrammed its machinery for sugar uptake while trying to grow under conditions where many of its native uptake pathways had got downregulated due to isobutanol stress. Also, this stress response had in some ways become constitutive because the evolved cells were now using these alternative pathways even in the absence of isobutanol.

### Gene transcript profiles in 4B4 culture

Apart from the constitutive changes in gene expression that occurred in the isobutanol evolved strain, we were also interested in looking at the inducible effect of isobutanol. Therefore, we analysed the RNA-sequencing data of the 40 g/l isobutanol evolved strain grown in the presence of isobutanol (4B4 culture). We could not however match the specific growth rates since this strain grew slowly and biomass build up was significantly lower, due to the presence of isobutanol.

The reads from RNA-seq were checked for their quality using FastQC and the analysis proceeded in a similar manner as done for the 4B0 culture. We found that the majority of genes showed a more enhanced response due to the presence of isobutanol in the 4B4 culture. Thus, most of the genes that were upregulated in 4B0 compared to wild type showed an even higher upregulation in 4B4 while the genes downregulated in 4B0 showed a correspondingly higher downregulation (Table 8). Interestingly, in spite of the evolution that had adapted the cells for higher tolerance, the cells retained a regulatory system which got upregulated due to the isobutanol stress. This enhanced response, which was over and above the constitutive changes in expression of the genes in 4B0 culture, clearly shows the important role of these differentially expressed genes in conferring isobutanol tolerance. However, the trend was bucked by a few genes that showed an opposite trend between 4B0 and 4B4 cultures like superoxide dismutase (LLNZ_02235), non-heme iron-binding ferritin (LLNZ_11895), universal stress protein uspA (LLNZ_10420), osmotically inducible protein C (LLNZ_00395) etc. which were found to be upregulated. Most of these were part of general stress regulons which possibly act in cascade and hence their effect on cellular physiology may be significant even with comparatively small changes in gene expression. Moreover, genes for some hypothetical proteins and putative transcriptional regulators whose roles and pathways are as yet unknown, were found upregulated in both 4B0 and 4B4 cultures, clearly demonstrating that they may have a role in alleviating isobutanol toxicity. We can therefore conclude that rather than a single gene or group of genes, this evolved isobutanol tolerant phenotype is the result of a comprehensive global reprograming of the cell.

**Table 8:** Genes that showed a higher degree of up and downregulation in 4B4 as compared to 4B0 culture.

S. No	Locus_tag	Annotation	LogFC 4B0	LogFC 4B4
1	LLNZ_06705	ATP phosphoribosyltransferase regulatory subunit	4.39	4.51
2	LLNZ_04305	Major head protein	4.21	6.16
3	LLNZ_11945	Arginine deiminase	3.54	4.57
4	LLNZ_05105	AcrR family transcriptional regulator	3.31	5.35
5	LLNZ_04595	Carbamoyl phosphate synthase small subunit	3.16	3.34
6	LLNZ_02125	NADH oxidase	3.12	4.17
7	LLNZ_03590	Heavy metal-(CdCoHgPbZn)-translocating P-type ATPase	2.98	9.07
8	LLNZ_00445	Putative HTH-type transcriptional regulator	2.9	3.24
9	LLNZ_08435	ABC transporter ATP-binding protein	2.9	4.41
10	LLNZ_09555	Multidrug resistance ABC transporter ATP-binding and permease protein	2.84	3.22
11	LLNZ_09250	Transcription elongation factor NusA	2.8	5.85
12	LLNZ_09770	Phosphate transporter ATP-binding protein	2.58	3.11
13	LLNZ_09995	Membrane-associated protein	− 2.66	− 4.25
14	LLNZ_12910	Glucose-6-phosphate 1-dehydrogenase	− 3.42	− 4.81
15	LLNZ_02015	Amino-acid permease lysQ	− 3.53	− 4.4
16	LLNZ_00010	DNA polymerase III subunit beta	− 2.72	− 3.36
17	LLNZ_03115	Adenine phosphoribosyltransferase	− 2.79	− 3
18	LLNZ_10005	Glutamine transport ATP-binding protein GlnQ	− 2.85	− 3.26
19	LLNZ_07555	Cobyric acid synthase	− 2.95	− 4.16
20	LLNZ_10650	Aminopeptidase C	− 3	− 3.74
21	LLNZ_03810	Cytidylate kinase	− 3.01	− 3.78
22	LLNZ_01190	Inosine 5′-monophosphate dehydrogenase	− 3.21	− 3.89

### Predicting the contribution of individual genomic mutations to the changed transcriptomic landscape leading to enhanced isobutanol tolerance

As stated earlier, even though the genome sequencing revealed a fairly small number of mutations, the RNA-seq analysis showed major changes in the evolved strain at the transcriptional level. It was therefore necessary to analyse this relationship and also how it finally led to the emergence of this tolerant phenotype. In the absence of data on the effect of individual mutations we can only hypothesize that the mutations in the regulatory sequences could have had some effect and may have contributed to this improved phenotype.

One such mutation was observed upstream of the LysR-family transcriptional regulator which controls a large family of prokaryotic regulatory proteins that includes the well-characterized CysB and MetR regulators^50^. These are involved in regulation of genes of sulphur metabolism and were found to be significantly upregulated (logFC = 4.96) in the evolved strain. Also, predominant among the upregulated genes were those involved in amino acid metabolism and membrane transport pathways. The gene O-acetylhomoserine sulfhydrylase (LLNZ_00455) known for its involvement in cysteine and methionine metabolism was the fourth most upregulated gene (logFC = 6.43) in the transcriptome of the evolved strain. This upregulation of the amino acid pathways, specifically that of cysteine and methionine metabolism, along with the genetic change upstream of the LysR transcriptional regulator is plausibly linked to improved tolerance. We also planned to look at the point mutation found in the upstream region of LysR family transcriptional regulator (as shown in Table 1 for 40 g/l isobutanol evolved strain) among few selected strains, where PCR product sequencing (of PCR amplicons generated using LysR_F and LysR_R primers) from genomic DNA of 19 g/l isobutanol evolved strain, 25 g/l isobutanol evolved strain and 32 g/l isobutanol evolved strain were performed (strains were selected randomly) (Supplementary Fig. S4A–C). However, Sanger sequencing (using LysR_F) results showed the absence of point mutation in sequenced PCR products from all the above evolved strains. Hence, the mutation in 40 g/l isobutanol evolved strain was indeed unique for LysR. However more experiments are required to firmly establish the claim that this mutation is one of the reasons behind this tolerant phenotype.

Similarly, one substitution mutation upstream of the gene ATPase, P-type (transporting), HAD superfamily, subfamily IC (LLNZ_13125) was found whose role is difficult to decipher, however the transcript of this gene was upregulated (logFC = 4.77) in the 4B0 culture. Members of this transporter superfamily catalyze cation uptake and/or efflux. Another mutation (insertion) was observed upstream of the phosphoglucosamine mutase gene (involved in peptidoglycan biosynthesis) which also showed upregulation (logFC = 2.6) in the 4B4 culture. However this response was clearly induced by isobutanol, since the same gene was found to be twofold downregulated in 4B0 culture. This shows that the presence of isobutanol triggered the upregulation of cell wall synthesis pathways in the evolved strain. Also, this insertion led to the restoration of a pseudogene by frameshift mutation into a sense polypeptide that showed sequence similarity with YbbR like protein of *L. lactis*. YbbR in known to have a role in acid-stress resistance in *S. aureus*^51^. The interaction of YbbR with its downstream gene phosphoglucosamine mutase has been observed in other bacteria^52^, hence, the combination of both events i.e. mutation as well as changed transcriptional stress response underscores the importance of this change for improved isobutanol tolerance. Another frameshift mutation was observed in the Phosphate transporter ATP-binding protein (LLNZ_09765) which led to its early truncation. This protein is a transmembrane protein involved in ATP dependent transport of inorganic phosphate. There are reports which show that single mutations, preferentially in genes involved in nucleotide metabolism and phosphate uptake, can result in elevated tolerance to multiple stresses^53,54^.

Another stop_gained mutation generated a variant of the d-alanine transfer protein DltD (LLNZ_06295) that causes the premature truncation of the protein involved in d-alanine esterification of lipoteichoic acid and wall teichoic acid. Interestingly its transcript was found to be upregulated (logFC = 2.13) in the 4B0 culture. However since the stop codon was significantly closer to the C-terminus, it is difficult to speculate on the functionality of the truncated polypeptide.

One missense_variant of peptidyl-prolyl cis–trans isomerase (LLNZ_08470) was observed, which however wasn’t altered at the transcriptional level. This mutation changed the third before last amino acid but again it is not possible to speculate on its significance, if any, and whether it altered the functionality of the protein. A Missense_variant of exodeoxyribonuclease V alpha chain (LLNZ_09895) involved in homologous recombination was also observed, which was also found to be upregulated (logFC = 2.19) in the 4B0 culture. Even more problematic were missense_variants of hypothetical proteins like LLNZ_06550 and predicted ATP-grasp enzyme (LLNZ_12940) which led to a one amino acid change in the polypeptide since clearly their roles are difficult to ascertain.

It is noteworthy that most of the mutations occurred in membrane proteins whose involvement hasn’t been highlighted in this work even though it’s logical to assume that the cell membrane would be the first barrier to exogenous isobutanol and its toxicity. The real problem is our incomplete knowledge of the linkages between genome and transcriptome and hence our inability to predict phenotype from changes in the genome sequence^55^.

We postulate that the other differentially expressed genes would be controlled by global transcriptional regulators, through cross regulatory mechanisms, since these too showed fluctuations in the isobutanol evolved strain. We thus see that a fairly complex synergistic response might have been generated with a few critical genomic mutations leading to a global cellular stress response which included changes in membrane transport, amino acid metabolism, sugar uptake and cell wall synthesis. Collectively these changes might be responsible for altering the cellular metabolism in ways such that it can tolerate the presence of isobutanol. However, the most direct method would be to pick up each of the mutations observed in the tolerant strain and individually incorporate them into the genome of the wild type strain in the hope of reverse engineering this tolerant phenotype. However since solvent tolerance is a multigenic trait we would need to construct and test a very large number of combinations of genomic modifications to arrive at the minimum set of mutations which are required for this phenotype. Also, different studies have identified different set of genes as contributors to alcohol tolerance even when the same stressors were used for ALE experiment. These discordances might have been caused by the differences in the experimental conditions^9^. For example, studies have shown^56,57^ that, depending upon information about target genes and metabolites obtained via omics analyses, it is possible to rationally engineer alcohol-tolerant bacterial strains.

Based on the above, several reports have noted a relationship between alcohol tolerance and specific amino acids^58,59^. We therefore adopted a similar approach (as discussed below) for our *Lactococcus* strain and borrowing from previous studies done on different microbes we tried to investigate the role of amino acids supplementation on growth of control (WT) and test (40 g/l isobutanol evolved strain) in the presence of different concentrations of isobutanol. These experiments could show that one of the mechanisms for improved isobutanol tolerance is enhanced utilization of amino-acids.

### Effect of amino-acid supplementation on growth of wild type *L. lactis* NZ9000 and evolved strain in presence and absence of isobutanol

Based upon the information provided by genome sequencing and transcriptomics, we narrowed down our approach to focus on the most affected upregulated category i.e. amino-acid biosynthesis pathways. Earlier reports have shown a connection between alcohol tolerance and specific amino-acids^12,13,58–60^. We therefore followed a similar strategy for investigating the effect of amino-acids supplementation (5 mM each) in improving the isobutanol tolerance of wild type NZ9000 strain (Supplementary Table S3) where an increase in biomass in the presence of amino-acids showed the ability of cells to cope with isobutanol toxicity. Combinations of amino-acids were selected based upon the gene expression profiles in RNA-seq data. We performed a similar study with the 40 g/l isobutanol evolved strain, to monitor its ability to tolerate isobutanol in presence of exogenously added amino-acids (5 mM each, Supplementary Table S3). Two different strategies for isobutanol addition were followed; in the first strategy isobutanol was added at the mid-exponential phase, while in the second case, isobutanol was added immediately after inoculation. The final biomass yields for all the above experiments are shown in Supplementary Table S3. The results clearly demonstrated the improved ability of wild type cells to grow in the presence of isobutanol (Supplementary Table S3) when the media was supplemented with an amino-acids cocktail. This confirms the role of amino-acids in protecting cells and allowing it to propagate even in the presence of a toxic organic solvent. However, the final biomass concentrations achieved depended upon the strategy of isobutanol addition with early addition of isobutanol being detrimental to growth (Supplementary Table S3). Thus the metabolic state of the cell plays an important role where actively growing cells tolerated isobutanol better than cells in lag phase. A similar trend was observed for the evolved strain as well. These experiments indirectly establish the upregulation of amino-acid biosynthesis pathways in 40 g/l isobutanol evolved strain to be an important factor in tolerance.

## Discussion

In the initial part of the ALE experiment, the seamless and rapid emergence of cells which could tolerate 28 g/l isobutanol demonstrates that there may be an intrinsic variability in the cell’s ability to tolerate solvent stress. Possibly a small sub-population of cells can tolerate higher concentrations of isobutanol and given sufficient time and the right environmental conditions it can overtake the dominant cell population. We labelled this phenomenon as adaptation to distinguish it from the spontaneous emergence of tolerant mutants in the latter part of the ALE experiments. This is a slower process and we therefore adopted a stop–go procedure while running the CSTR to prevent loss of these beneficial mutations. That fact that we had to stop the CSTR multiple times, points to the additive effect of the accumulated mutations leading finally to the hyper tolerant strain.

**Figure 5:**
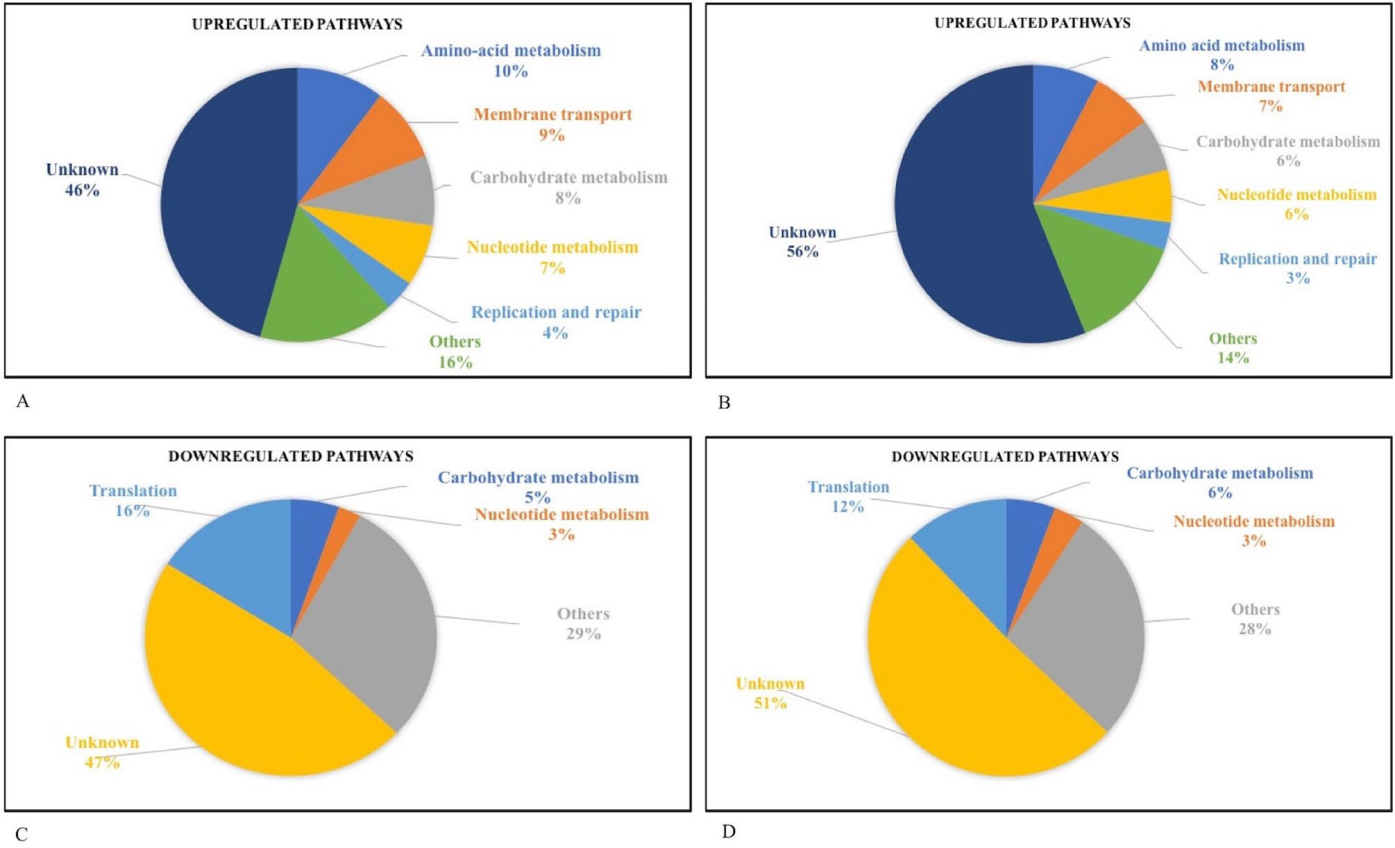
(**A**) Pie chart showing the upregulated pathways (logFC ≥ 2.5). Amino-acid metabolism and membrane transport pathways were maximally upregulated among the differentially expressed genes. (**B**) Pie chart showing the upregulated pathways (logFC ≥ 1.5). Amino-acid metabolism and membrane transport pathways remained maximally represented in the upregulated genes even when the gene numbers were increased (cut off was decreased). (**C**) Pie chart showing the downregulated pathways (logFC ≤ −2.5). Translation and carbohydrate metabolism pathways were maximally downregulated among the differentially expressing genes. (**D**) Pie chart showing the downregulated pathways (logFC ≤ −1.5). Translation and carbohydrate metabolism pathways remained maximally represented among the downregulated pathways even when the gene numbers were increased (cut off was decreased).

While we do now have a strain with the desirable characteristics of high isobutanol tolerance, problems remain. The most critical of these is the development of resistance to a range of antibiotics which makes the task of genetic manipulation harder. This is possibly due to the enhanced activity of efflux pumps which ensures cell survival in the presence of toxic compounds, whether it is antibiotics or isobutanol. The other is the wide range of changes that characterize the transcriptomic landscape of the evolved strain. We were hoping that the evolutionary response would be limited to a few pathways, as observed in bacteria during prolonged exposure to heavy metals where only the oxidative stress response is triggered^61,62,63^. However, our study demonstrates that a synergistic action of multiple genes is required for the cell to develop solvent tolerance, precluding any attempt to reverse engineer this tolerant phenotype. A related problem is the possibility of accumulating drift mutations, those that may not have any role in providing tolerance. To investigate this, we looked at the pathways with logFC ≥ 2.5 and observed that the amino acid metabolism and membrane transport pathways were maximally upregulated (Fig. 5A). We broadened our search by reducing logFC cut-offs to ≥ 1.5, and again observed upregulation in the same category of pathways (Fig. 5B). The fact that even when we look at different degrees of upregulation, it remains limited to specific pathways demonstrates their essential role in isobutanol tolerance. This data corroborates the observed downregulation of CodY, a transcriptional repressor that leads to upregulation of nitrogen metabolism and also the upregulation of TetR which triggers the upregulation of membrane transporters and efflux pumps. This reprogramming of the cellular machinery is critical to survival since it allows the cells to grow on amino acids, which serve as an alternative carbon and energy sources when the glucose uptake pathways are compromised. Also, the upregulation of a large numbers of membrane transport pathways might help the cell reduce its intracellular isobutanol concentration through the efflux pumps. Apart from above categories, unknown pathways and other metabolic pathways also showed major fluctuations whose contribution to the isobutanol tolerant phenotype is difficult to assess.

When we look at the downregulated pathways (logFC ≤  − 2.5), translation and carbohydrates metabolism genes were overrepresented (Fig. 5C). If we broaden our logFC cutoffs to ≤  − 1.5, the same pathways remained maximally overrepresented (Fig. 5D). Clearly the uptake of sugars gets compromised leading to a cascade effect on carbohydrate metabolism. This in turn reduces energy levels and may lead to the slowdown of energy demanding processes, like translation. These changes thus represent the logical counterpart of the upregulated response and taken together allow the cell to survive in a toxic environment. As in the upregulated case, here also we observed that many unknown and unrelated genes showed significant downregulation and their role in isobutanol tolerance is difficult to ascertain.

An indirect way to establish the criticality of these genes was to look at the transcriptome of these evolved cells when isobutanol was present in the medium. Interestingly we observed that the change in expression of most of these genes got exacerbated, with upregulated genes getting further upregulated and downregulated genes showing higher downregulation. Clearly these changes were essential for tolerance since the presence of isobutanol further enhanced these effects. The evolved cell is thus ‘primed’ for tolerance by incorporating changes in its transcriptional state even in the absence of isobutanol.

## Material and methods

### Bacterial strains and primers

The bacterial strains and primers used in this study are listed in Supplementary Table S1 and S2.

### Static flask study

The glycerol stock of *L. lactis* NZ9000 was streaked in GM17 (M17 broth from HiMedia with exogenous addition of 5 g/l glucose) agar plate and kept at 30 °C incubator for 18–24 h. Single isolated colony was inoculated in 10 ml GM17 media and grown in static flask at 30 °C for 18–24 h. The primary culture was inoculated in 100 ml GM17 media (volume adjusted accordingly) containing different concentration (0–20 g/l) of isobutanol. The optical density (OD_600_) was measured using spectrophotometer and recorded every hour. The specific growth rate was calculated from the linear range of exponential growth.

### Continuous stirred tank reactor set up and operation

The media was manually modified from the composition of standard M17 media containing 2.5 g/l Yeast extract, 5 g/l Soybean casein digest, 5 g/l Beef extract, 2.5 g/l Tryptone, 0.25 g/l Magnesium sulfate with exogenous addition of 5 g/l Glucose. Bioreactor was started with the modified GM17 media with an initial concentration of 8 g/l isobutanol. The pH of the reactor was maintained at 6.5 by continuous buffering through 2 N Hydrochloric acid and 5 N Sodium Hydroxide. The anaerobic conditions were maintained by continuous flow of 50 ml/min nitrogen in the reactor and temperature was maintained at 30 °C.

Continuous culture was carried out in an Electrolab, FerMac360 reactor. Modified GM17 media was used in culture vessel as well as in the feed. The working volume in the reactor vessel was maintained to 730 ml. The continuous feed with isobutanol was supplied in the reactor and outlet was maintained at same flow rate so that the total volume remains same inside the reactor. Initially the feed rate was maintained at 115 ml/h with dilution rate of 0.159. As the experiment progressed the feed rate was changed according to specific growth rate. Biomass was monitored continuously by measuring and recording OD_600_. The isobutanol was gradually increased from (8–40 g/l) in the feed tank to maintain the continuous selective pressure in the reactor vessel, which causes the enrichment of isobutanol tolerant cells.

### Polymerase chain reaction (PCR) of 16S rRNA gene

The protocol for 16S rRNA amplification was adapted from^36^ with slight modifications in which we have used the forward primer specific for subsp. *cremoris* and reverse primer specific for species of *L. lactis.* PCR amplification reaction mixture (20 μl) contains 1 μl template DNA, 0.1 μl (100 nmol) of both forward (CreF) and reverse primer (LacreR) from 20 μM stock, 2 μl 10 × Taq buffer, 0.1 μl 2 mM dNTP, 0.2 μl Taq polymerase and 16.6 μl double distilled water. The reaction mixture was cycled 30 times in a thermocycler using the following conditions; 94 °C for 5 min, 94 °C for 30 s, 58 °C for 30 s, 72 °C for 30 s and again 72 °C for 5 min. The PCR products were analyzed by electrophoresis using 1% agarose gels in tris–acetate-EDTA (TAE) buffer^64^.

### Genome sequencing and mutation discovery

Genomic DNA of the 4B0 culture was purified manually as: 2 ml GM17 broth was inoculated from the single isolated colony from the streaked GM17 agar plate and culture was grown for minimum of 18 h at 30 °C incubator under static conditions and next day cells were centrifuged at 18,000×*g* for 10 min. The supernatant was discarded and pellet was resuspended in 850 μl of TEN buffers (10 mM Tris-pH 8, 1 mM EDTA, 0.1 M NaCl) and 150 μl of lysozyme. Mixture was incubated at 37 °C water bath for 30 min. 115 μl of 10% SDS and 1 μl of proteinase K was added and again incubated at 37 °C for 1 h. Then 130 μl of 5 M NaCl and 750 μl of chloroform isoamyl alcohol (24:1) were added and shaken vigorously. The mixture was centrifuged at 18,000 g for 5 min. The supernatant was removed and transferred into 2 ml vial. 700 μl of propan-2-ol was added and shaken vigorously. This was again spun at 18,000×*g* for 5 min. The supernatant was discarded and pellet was washed with 1 ml of 70% ethanol and spun at 18,000×*g* for 5 min. The pellet was dried and dissolved in 150 μl of TE buffer and 1 μl of RNase A (10 mg/ml) and the quality was checked by DNA electrophoresis and NanoDrop 1000 (Thermo Fisher Scientific) analysis. Genome sequencing was performed by AgriGenome (Cochin), India. The procedure described briefly: Genomic DNA was fragmented and paired end library (2 × 100bpPE) was prepared. The DNA fragment libraries was validated by tapestation and sequenced on illumine HiSeq 2500 platform. We opted for 30 × coverage data output for this sample. The Bioinformatics analysis was also performed in their facility which involves following steps: The reads were checked for their quality and sequence read was trimmed where necessary to only retain high quality sequence for further analysis. The low-quality sequence reads were excluded from the analysis, the paired end reads were aligned to the available reference genome of the NZ9000. The alignment was performed using BWA mem (Version-0.7.12) program with the default parameters.

The aligned reads are first sorted using Picard tool SortSam command and then the read duplicates were removed using Picard Mark Duplicates command. Samtools was used to identify single nucleotide variants (SNPs) and short Indels. After calling the variants, they were further filtered in order to retain good quality (depth, variant score and others) variants. The identified variants were annotated using snpEff tool and their in-house Perl program.

### Confirmation of stability of evolved phenotype of selected strains

The selected isobutanol evolved strains from CSTR were streaked on individual GM17 agar plates along with the native NZ9000 (control) without isobutanol (selection pressure removed). A single colony was picked and inoculated in the primary media without isobutanol (selection pressure was again removed). This ensured the removal of selection pressure for approximately 15–20 generations while the cells were growing on the plate as well as primary culture without isobutanol. Inoculum from the primary culture was then inoculated in the secondary medium with different concentrations of isobutanol (different percentages of isobutanol was used for different percentages of isobutanol evolved strains as discussed in results) to confirm the stability of the tolerant phenotype. Biomass was monitored in the duration of 2 h by measuring and recording OD_600_. The specific growth rate was calculated from the linear range of exponential growth.

### Sample preparation for SEM and cell sizes calculation

Glycerol stocks of the native NZ9000 and the isobutanol evolved strains were streaked in the GM17 agar plates and kept at 30 °C incubator for 18–24 h. Single colonies from each plate were inoculated in the GM17 broth with and without isobutanol. The cultures were grown anaerobically in screw cap bottles in 30 °C incubator for 24–48 h. (especially the isobutanol evolved strains whose doubling time has increased). 5 × 10^8^ cells were taken from each strain and harvested at 8,000 rpm for 5 min and washed with 1 × phosphate buffered saline (PBS) buffer (n = 5) and fixed with 500 μl of 2.5% glutaraldehyde at room temperature for 2 h and then samples were kept at 4 °C for overnight incubation. Next day fixative was discarded and again cells were washed with 1 × PBS (n = 5) and samples were given to advanced instrumentation research facility (AIRF), JNU for further preparation and analysis. For calculations of the average cell sizes, SEM was performed using cultures from biological duplicates and sizes were calculated by counting 20 cells in each culture/strain obtained at same magnification using ImageJ software.

### Membrane potential analysis

The fluorescent dye DiSC_3_ was used to monitor the membrane potential of the whole cells. Approximately 30 ml of 2 × 10^8^ cells/ml were harvested at 2,800×*g* for 10 min and washed twice with 50 mM potassium phosphate buffer (KPi) pH7. The remaining protocol was followed as described for *L. monocytogenes*^65^. The experiments were performed in Cary Eclipse fluorescence spectrophotometer and fluorescence was measured with an excitation and emission wavelength of 643 nm of 666 nm (both with a 5-nm band pass).

### RNA isolation and whole cell transcriptome sequencing

Total RNA was isolated by slight modification of the method described for *L. lactis*^66^. Cells from the late-log phase were snap frozen and harvested at (5,000×*g*, 10 min, 4 °C) for the native and the evolved strains. Cell pellets were resuspended in 500 μl of ice-cold TE buffer pH 7.5 and transferred to ice-cold 2 ml microfuge tube (Eppendorf), after which 500 μl phenol/chloroform mixture (1:1), 30 μl 10% SDS, 30 μl 3 M sodium acetate (pH 5.2), and 500 mg glass-beads (150–212 μm, Sigma-Aldrich, St. Louis, USA) were added. The mixture was vortexed for 4 min at maximum speed and then 400 μl of chloroform isoamyl alcohol (24:1) was added. The mixture was spun at 10,000×*g* at 20° for 2 min. The supernatant was transferred to fresh microfuge tube and equal volume of RNAzol (Sigma) was added and steps were followed according to manufacturer’s protocol. The total RNA concentration and quality was determined by Agilent bioanalyzer 2,100 expert. The good quality and quantity of total RNA were given to AgriGenome (Cochin), India for library preparation and RNA-sequencing.

### Transcriptome analysis

Data was analyzed by reference genome based analysis using the reference genome of *L. lactis* NZ9000 *Lactococcus_lactis*_subsp_cremoris_nz9000.ASM14320v1.dna.toplevel.fa.gz (Downloaded from Ensembl), abundance measurements and differential expression was performed using RSEM^67^ and edgeR^68^. Pathway enrichment was done using Kyoto encyclopedia of genes and genomes (KEGG)^69^.

### RT-qPCR

RT-qPCR was carried out to validate the RNA-seq results. The gene-specific primers used in this study are shown in Supplementary Table S2, and their specificity was confirmed by melting curve analysis. Gene *rpmf* (encoding 50S ribosomal protein L32) was used as the internal reference because it has shown higher transcriptional stability under isobutanol stress conditions in RNA-seq data analysis. RT-qPCR analysis were performed with the same RNA samples used for RNA-seq analysis. Genomic DNA contamination was again ruled out by setting PCR with any of the RT-qPCR primers using total RNA as template. For cDNA synthesis, 1 μg of total RNA was reverse transcribed using RevertAid first strand cDNA synthesis kit (Thermo). The efficiency of cDNA synthesis was validated by using different cDNA dilutions in initial standard PCR reactions. The qPCR was conducted by using KAPA SYBR FAST qPCR Master Mix (2X) Kit and 2^-ΔΔ*CT*^ method^70^ was used for relative quantification of log fold changes.

### Static flask study using amino-acid supplementation

The glycerol stock of *L. lactis* NZ9000 and 40 g/l isobutanol evolved strain was streaked in GM17 (M17 broth from HiMedia with exogenous addition of 5 g/l glucose) agar plate and kept at 30 °C incubator for 18–24 h. Single isolated colony was inoculated in 20 ml GM17 media and grown in static flask at 30 °C for 18–24 h. The secondary culture was inoculated in 10 ml GM17 media (volume adjusted accordingly) with and without amino-acid supplementation along with and without isobutanol as indicated in Supplementary Table S3. Stock of 200 mM amino-acids cocktail containing (Tryptophan, tyrosine, phenylalanine, cysteine, methionine, aspartic acid, arginine, histidine, valine, leucine, isoleucine) was prepared and 5 mM (final concentration) was added in secondary media where indicated. The optical density (OD_600_) was measured using spectrophotometer and recorded at 0 and 24 h.

## Supplementary information

Supplementary file

## Data Availability

The RNA-seq data has been deposited in the Gene Expression Omnibus database at national center for biotechnology information (NCBI) (GEO: https://www.ncbi.nlm.nih.gov/geo/query/acc.cgi?acc=GSE107996) under the Accession Number GSE107996. Genome sequencing data has been deposited to SRA with accession PRJNA492719 and the draft genome has been deposited to NCBI under the GenBank accession CP032526. All other information regarding data generated and materials used in the present study are presented in the paper and supplementary files.
