# Supplementary material for: Genomics and transcriptomics analysis reveals the mechanism of isobutanol tolerance of a laboratory evolved Lactococcus lactis strain
Source: Sci Rep. 2020 Jul 2;10:10850. doi: 10.1038/s41598-020-67635-w (PMC7331579; doi:10.1038/s41598-020-67635-w)

Genomics and transcriptomics analysis reveals the mechanism of isobutanol tolerance of a laboratory evolved *Lactococcus lactis* strain

Jaya Anand Gupta[a*], Sagar Thapa[a], Madhulika Verma[b], Ritu Som[a], Krishnajyoti Mukherjee[a]

[a]Bioprocess and Biosystems Engineering Laboratory, School of Biotechnology, Jawaharlal Nehru University, New Delhi, 110067, India

[b]School of Computational and Integrative Sciences, Jawaharlal Nehru University, New Delhi, 110067, India

Email addresses:

JAG: jaya25_sbt@jnu.ac.in

ST: thapasagar001@gmail.com

MV: madhul27_sit@jnu.ac.in

RS: rituthakur345@gmail.com

KJM: kjmukherjee@jnu.ac.in

* Corresponding author

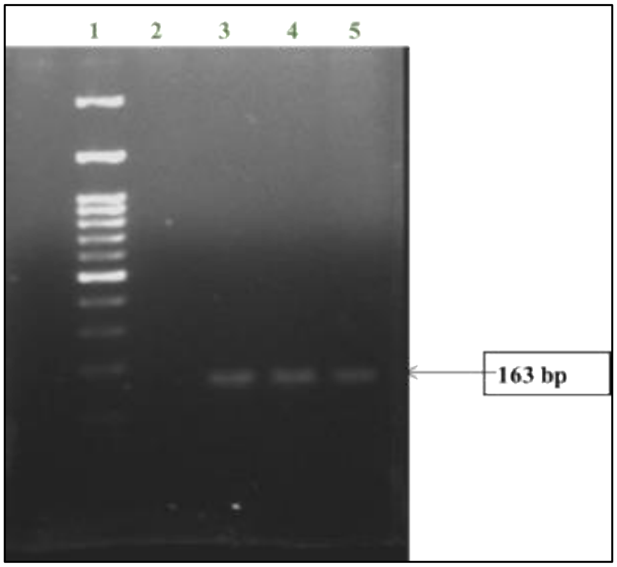

Supplementary Fig.1. Confirmation of the strain genotype using PCR. Lane 1-100 bp ladder, Lane 2- Negative control, *E. coli* genomic DNA as template, Lane 3- PCR amplicon from native NZ9000 genomic DNA, Lane 4- Sample from CSTR with 28g/l isobutanol tolerance, Lane 5- Sample from CSTR with 40g/l isobutanol tolerance. Image acquisition parameters were: Exposure time- 1.321 seconds, Light mode- reflective, Size mode- relative. Image pixels were: X: 1360, Y: 1024 and Pixel size (um) was X: 100.0, Y: 100.0

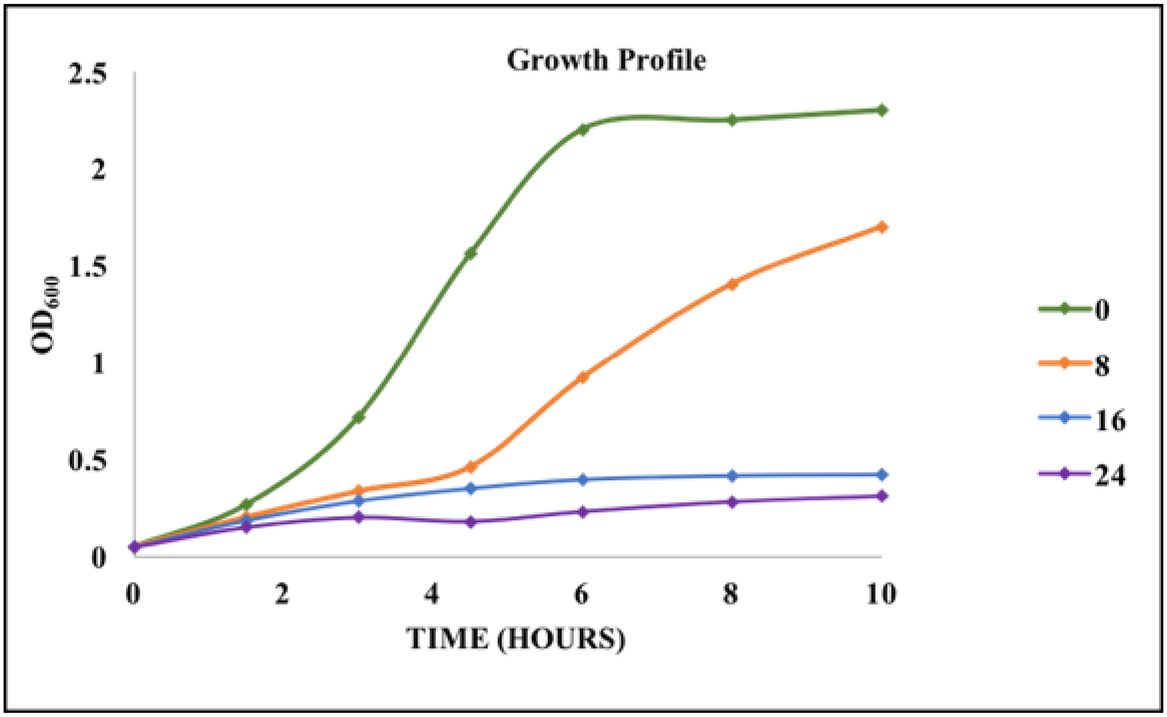

(A)

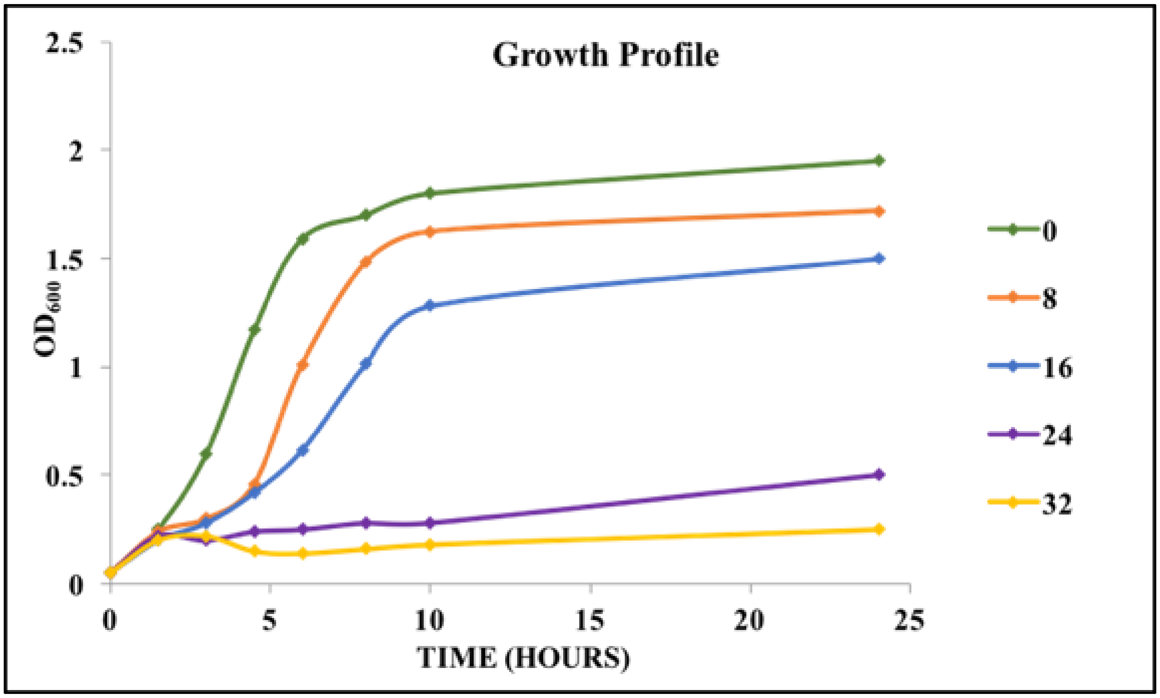
Growth Profile
OD600
TIME (HOURS)
0
8
16
24
32

(B)

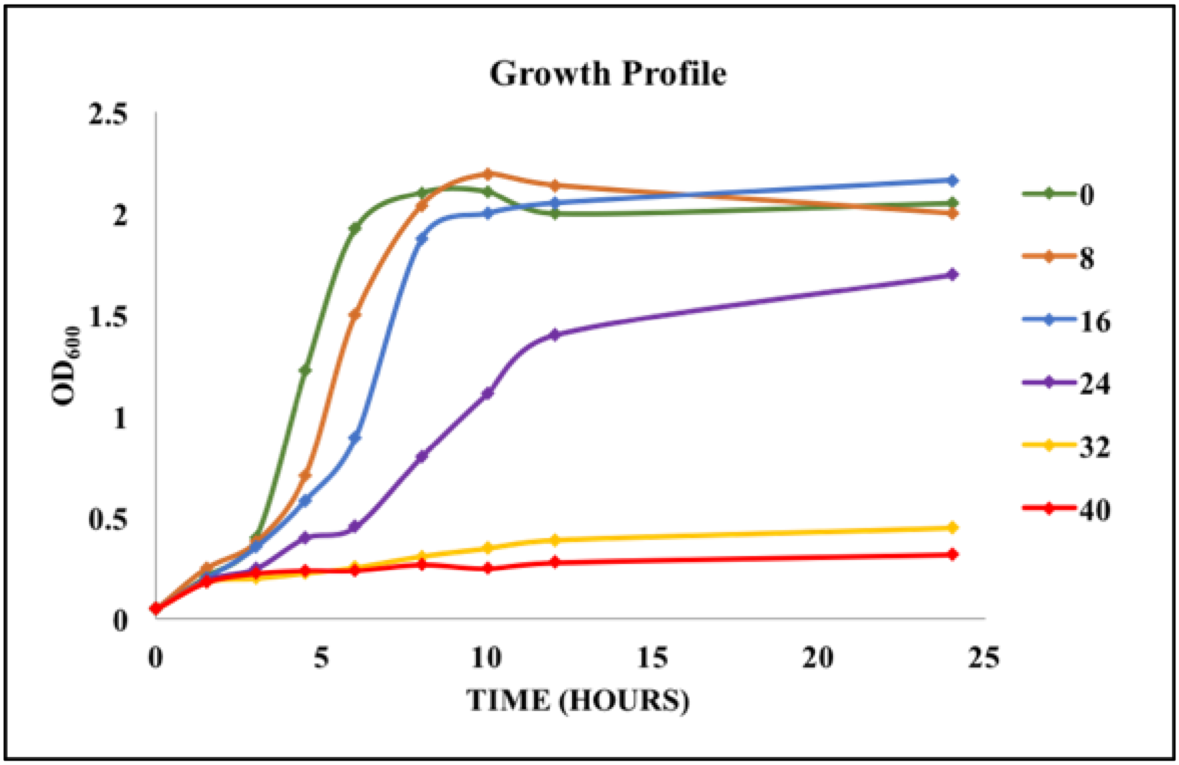
Growth Profile
OD600
TIME (HOURS)
0
8
16
24
32
40

(C)

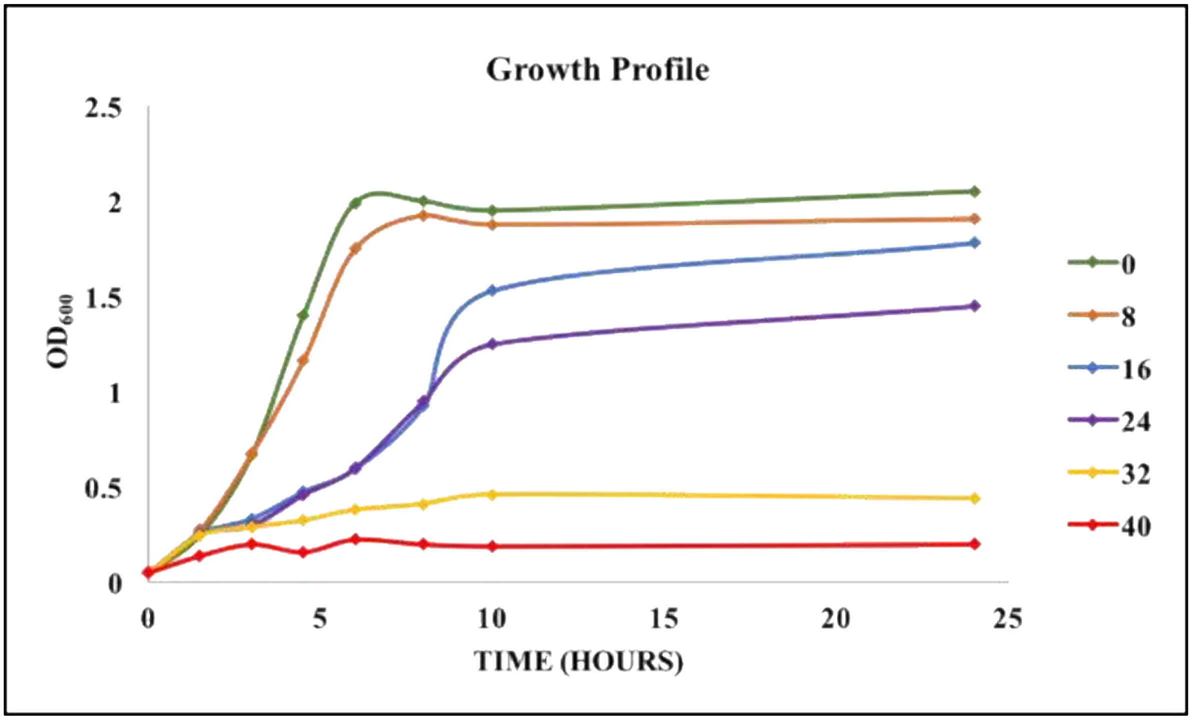

(D)

Supplementary Fig.2. (A-D). Static flask study for stability confirmation of the selected isobutanol tolerant strains (23g/l, 30g/l, and 40g/l) including the native NZ9000 in presence of 0,8,16,24,32 and 40g/l isobutanol). A) Native NZ9000 strain was unable to tolerate above 8g/l isobutanol as the specific growth rate falls to zero. B) The 23g/l evolved strain could efficiently tolerate 16g/l isobutanol, however the growth rate was less when grown at 24g/l isobutanol. C) The 30g/l evolved strain could well tolerate 24g/l isobutanol, however, at 32g/l the growth rate was almost similar to 40g/l isobutanol containing media. D) The 40g/l evolved strain was also able to tolerate 40g/l isobutanol but biomass build up was less as the substrate uptake rates were compromised.

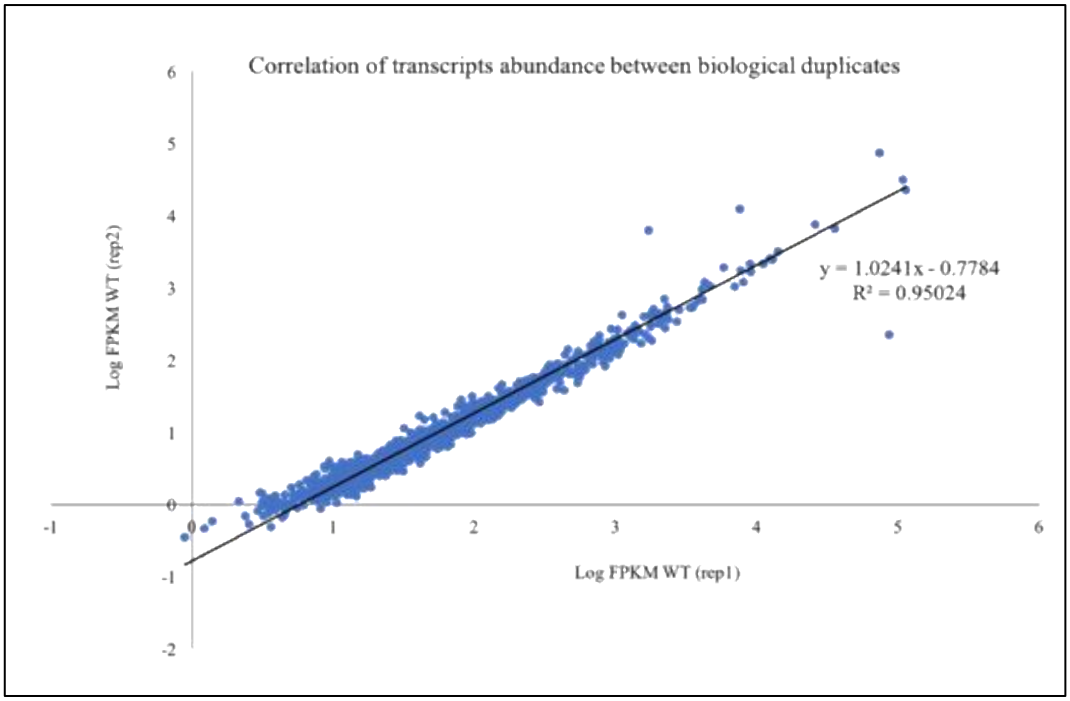

Supplementary Fig.3. Scatter plot. Log FPKM comparisons between biological duplicates of native NZ9000 (control) samples.

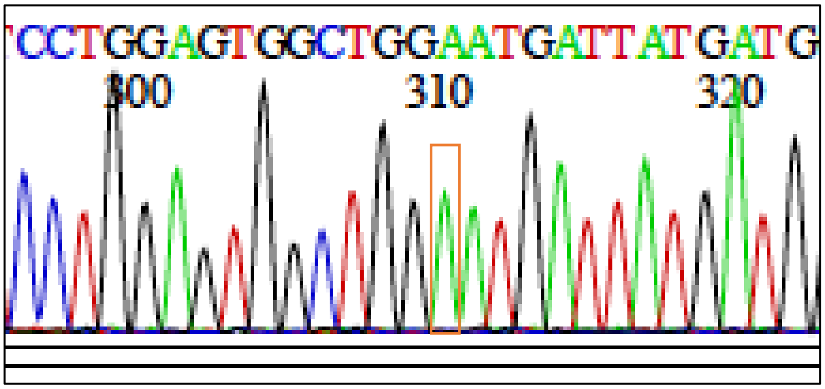

(A)

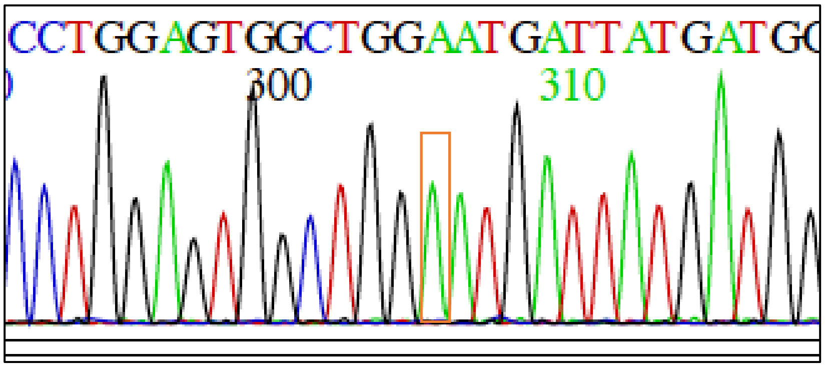

(B)

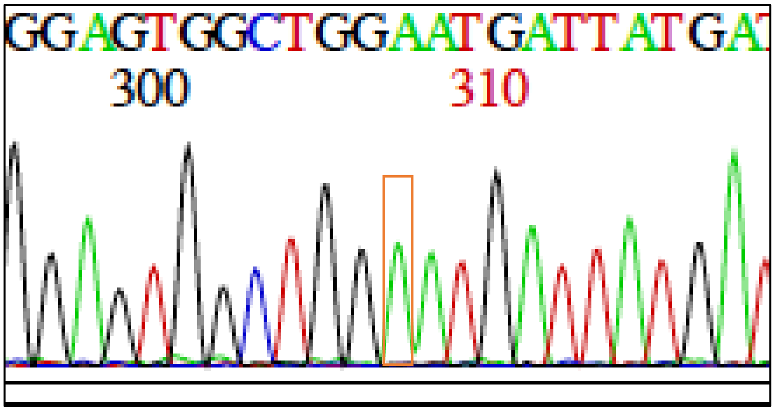

(C)

Supplementary Fig.4. (A-C). Sequencing chromatograms. A) PCR product sequencing results using LysR_F of 19 g/l isobutanol tolerant strain. B) 25 g/l isobutanol tolerant strain. C) 32 g/l isobutanol tolerant strain. In all the above three strains we have scanned the upstream region (genome position- 391179-391580) of *lysR* gene.The red rectangle in the chromatogram shows the intact A nucleotide at position 391489 in all the above evolved strains which got mutated to G in 40 g/l isobutanol tolerant strain as shown in Table 1.

Table S1: Strains Used and Evolved in this Study

**Strains**	**Description**
WT	Native NZ9000
23 g/l isobutanol evolved	Isobutanol evolved mutant from CSTR that could tolerate 23 g/l isobutanol

30 g/l isobutanol evolved	Isobutanol evolved mutant from CSTR that could tolerate 30 g/l isobutanol
40 g/l isobutanol evolved	Isobutanol evolved mutant from CSTR that could tolerate 40 g/l isobutanol

Table S2: Primers Used in this Study

**Primer name**	**Primer sequence from 5' to 3'**
CreF	GTGCTTGCACCGATTTGAA
LacreR	GGGATCATCTTTGAGTGAT
Sucrose-specific PTS system IIBC component_F	TCCGCTTTTTCCTTAGCATCTTT
Sucrose specific PTS system IIBC component_R	GGGTGCTTATGTGGGTGGAT
Carbon starvation protein A_F	TTCTGCTTGGGCAGTCTTTT
Carbon starvation protein A_R	TATAGCCCATCACCCACCAT
Multidrug resistance ABC transporter ATP-binding and permease protein _F	TTTCCTGTCAGCTCAAGAAAAAC
Multidrug resistance ABC transporter ATP-binding and permease protein _R	TGACACAAATTCTTTTACTTTTGGA
Pyruvate kinase_F	TGCTGCAGAAGCTATGGATG
Pyruvate kinase_R	CAACCAAACCAGAAGCCAAT

Non-heme iron binding ferritin_F	CCCCAAGAGCAGTTGTGAAT
Non-heme iron binding ferritin_R	TTGGATGAATTTGCAGAACG
Rpmf_F (Reference gene)	GAACTTTGCGGCCTTTGTAG
Rpmf_R (Reference gene)	CCTGCACGTCACACTTCATC
LysR _F	AATCATGTTTTACCAAAAGA
LysR_R	TACCGACAAAAGTGACAATT

Table S3. Effect of amino-acids supplementation in investigating tolerance of wild type and 40 g/l isobutanol evolved strain in presence of different isobutanol concentrations.

**Time (Hrs.)**	**OD_{600} WT(GM17)**	**OD_{600} WT(GM17+5 mM AA)**	**OD_{600} WT(GM17+5 mM AA+10 g/l IsoB)**	**OD_{600} WT(GM17+5 mM AA+15 g/l IsoB)**	**OD_{600} WT(GM17+5 mM AA+20 g/l IsoB)**	**OD_{600} WT(GM17+5 mM AA+25 g/l IsoB)**
0	0.4 ±0	0.4 ±0	0.4 ±0	0.4 ±0	0.4 ±0	0.4 ±0
24	2.7 ±0.14	3.8 ±0.14	3.5 ±0.07	3 ±0.14	2.9 ±0.07	2.8 ±0.14

OD_{600} values of wild type cells cultured with and without amino-acid supplementation when respective isobutanol (IsoB) concentrations were added to mid-exponentially growing cultures. AA represents amino-acids.

Time (Hrs.)	OD_{600} 4B0(GM17)	OD_{600} 4B4(GM17+40 g/l IsoB)	OD_{600} 4B0(GM17+5mM AA)	OD_{600} 4B4(GM17+5mM AA+40 g/l IsoB)
0	0.4 ±0	0.4 ±0	0.4 ±0	0.4 ±0
24	2.4 ±0.07	1.5 ±0	3.2 ±0.14	2.2 ±0.07

OD_{600} values of 40 g/l isobutanol evolved strain cultured with and without amino-acid supplementation when 40 g/l IsoB was added to mid-exponentially growing cultures. 4B0 represents when grown in absence of isobutanol and 4B4 represents when grown in presence of 40 g/l isobutanol.

Time (Hrs.)	OD_{600} WT(GM17)	OD_{600} WT(GM17+10 g/l IsoB)	OD_{600} WT(GM17+5mM AA)	OD_{600} WT(GM17+5mM AA+10 g/l IsoB)	OD_{600} WT(GM17+5mM AA+15 g/l IsoB)	OD_{600} WT(GM17+5mM AA+20 g/l IsoB)	OD_{600} WT(GM17+5mM AA+25 g/l IsoB)
0	0.4 ±0	0.4 ±0	0.4 ±0	0.4 ±0	0.4 ±0	0.4 ±0	0.4 ±0
24	2.5±0.07	0.5 ±0	3.8 ±0.07	2 ±0.14	1.2 ±0.07	0.8 ±0.07	0.6 ±0.07

OD_{600} values of wild type cells cultured with and without amino-acid supplementation when respective IsoB concentrations were added immediately after inoculation.

Time (Hrs.)	OD_{600} 4B0(GM17)	OD_{600} 4B4(GM17+40 g/l IsoB)	OD_{600} 4B0(GM17+5mM AA)	OD_{600} 4B4(GM17+5mM AA+ 40 g/l IsoB

0	0.4 ±0	0.4 ±0	0.4 ±0	0.4 ±0
24	2.5 ±0	0.5 ±0.07	3.1 ±0.07	1.3 ±0.07

OD_{600} values of 40 g/l isobutanol evolved strain cultured with and without amino-acid supplementation when 40 g/l IsoB was added immediately after inoculation. 4B0 represents when grown in absence of isobutanol and 4B4 represents when grown in presence of 40 g/l isobutanol.

Supplementary Fig. 1. Confirmation of the strain genotype using PCR (Full length gel).

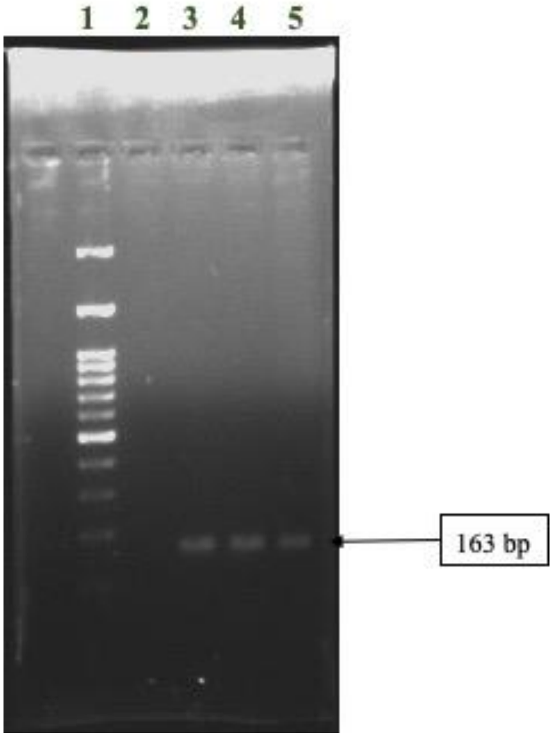

Supplement: Supplementary file 1 — Supplementary file [file 41598_2020_67635_MOESM1_ESM.pdf]
